# Continuous-flow-enabled intensification in nitration processes: a review of technological developments and practical applications over the past decade

**DOI:** 10.3762/bjoc.21.132

**Published:** 2025-08-26

**Authors:** Feng Zhou, Chuansong Duanmu, Yanxing Li, Jin Li, Haiqing Xu, Pan Wang, Kai Zhu

**Affiliations:** 1 National & Local Joint Engineering Research Center for Deep Utilization Technology of Rock-salt Resource, Faculty of Chemical Engineering, Huaiyin Institute of Technology, Huai’an 223003, P. R. China; 2 China Construction Industrial & Energy Engineering Group Huanghe Construction Co., Ltd., 7 Yandong Xinlu, Lixia District, Jinan 250000, P. R. China; 3 China Construction Industrial & Energy Engineering Group Co., Ltd., Nanjing 210023, P. R. China

**Keywords:** continuous-flow, kinetics, nitration, optimization, scale up

## Abstract

Flow chemistry technology has demonstrated significant potential in advancing the green transformation of the chemical industry while enhancing inherent process safety. Safety, cost-effectiveness, and operational efficiency serve as pivotal drivers for advancing flow chemistry in nitration processes. This review provides a comprehensive analysis of the continuous-flow nitration technology – a process historically recognized as one of the most hazardous industrial operations – focusing on its technological advancements in process design, reaction kinetics characterization, and practical implementation over the past decade. Detailed discussions encompass system configuration strategies, critical process parameters and operating ranges, performance evaluation metrics, universal methodologies for kinetics analysis, safety assessment protocols, and scale-up approaches. The presented content aims to offer actionable guidance for researchers and engineers engaged in the development of continuous-flow nitration systems.

## Introduction

**Figure 1:**
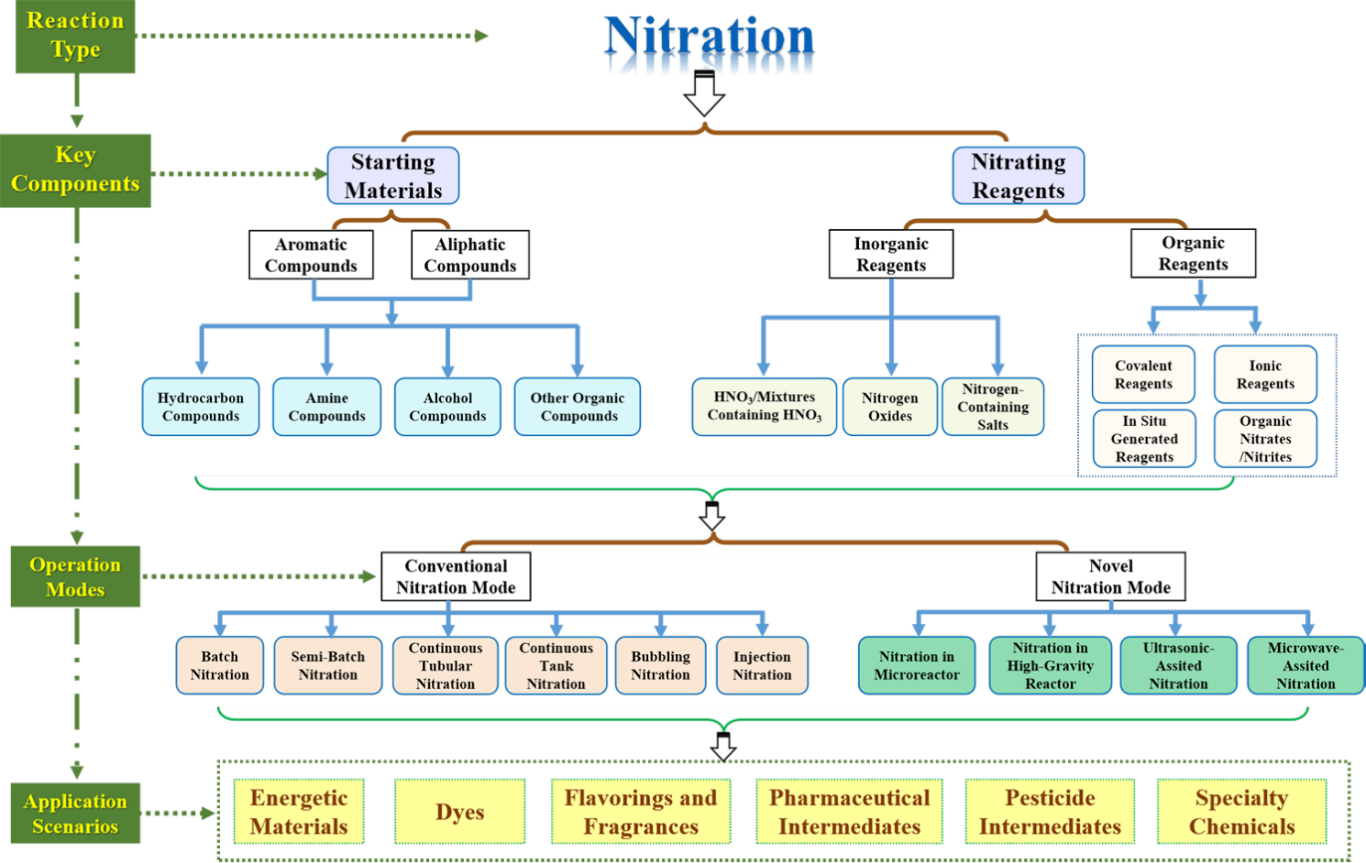
Three key dimensions of a complete nitration process.

Nitro compounds hold an extremely important position in the field of organic chemistry, mainly because they are easily obtainable and can be converted into a variety of functional groups. Nearly two centuries ago Ascanio Sobrero [1] prepared explosiv nitroglycerin through a nitration reaction using a mixture of nitric and sulfuric acids at low temperatures. Over the subsequent century and beyond the application scope of nitration has been expanded dramatically, and the number of nitrogen-containing compounds continuously increased. These compounds find broad applications in synthesizing energetic materials, dyes, flavorings and fragrances, pharmaceutical and pesticide intermediates, as well as specialty chemicals [2]. As delineated in Figure 1, a complete nitration process encompasses three key dimensions: the selection of starting materials and nitrating reagents, the implementation of the nitration reaction, and the specific application scenarios of the nitration products.

The nitration reaction serves as a pivotal synthetic pathway for nitro compound production, fundamentally involving the interaction between organic substrates and nitrating reagents. The intrinsic kinetics of nitration processes are governed by the synergistic interplay between the substrate’s molecular architecture and nitrating reagent reactivity. Distinct substrate classifications necessitate tailored nitrating reagent selections, giving rise to fundamentally distinct mechanistic pathways. This reactivity is exemplified by the comparative kinetic profiles: N-nitration of amines and O-nitration of alcohols – facilitated by accessible lone electron pairs – exhibit markedly higher kinetic feasibility than aliphatic C-nitration in saturated carbon frameworks [3]. Following determination of substrate–nitrating agent combinations, reactor system specification emerges as the critical engineering decision. Nitration reactor selection – encompassing batch, semi-batch, continuous tubular, continuous tank, bubbling and injection nitration reactor – directly governs thermodynamic control, product distribution, process scalability, and quality assurance [4–5]. Batch systems remain prevalent for low-throughput fine chemicals due to their operational flexibility, whereas continuous-flow configurations demonstrate superior safety and efficiency. While the synthetic significance of nitration is underscored by the broad utility of nitro derivatives, persistent technical challenges including regioselectivity control, over-nitration phenomena, and substrate oxidation side reactions coexist with engineering bottlenecks requiring optimization in process safety and scale-up strategies. Contemporary research endeavors prioritize two synergistic pathways: (i) pioneering alternative nitration pathways via innovative chemical approaches, particularly through novel nitrating agent development, and (ii) refining existing synthetic processes through process intensification strategies.

Nitric acid (HNO_3_) and its binary systems with sulfuric acid (HNO_3_/H_2_SO_4_) remain the industry-standard nitrating systems, particularly for the nitration of aromatic substrates. Motivated by environmental, safety, and efficiency concerns, researchers have explored various types of inorganic and organic nitrating reagents to improve reaction performance through innovative chemical approaches. Patra et al. comprehensively reviewed the development and applications of organic nitrating reagents in nitration reactions up to 2021, and summarized the characteristics of the main organic nitrating reagents discussed in their work [6]. Yang et al. developed 5-methyl-1,3-dinitro-1*H*-pyrazoleas a controllable nitronium ion source for mild, selective (hetero)arene nitration. This method achieves condition-controlled selective mono- or di-nitration and facilitates late-stage C–H nitration of biorelevant molecules, with DFT/mechanistic studies revealing synergistic 'nitro effect' and 'methyl effect' underlying the reactivity [7]. Jia et al. developed dinitro-5,5-dimethylhydantoin (DNDMH) as an efficient arene nitration reagent, demonstrating broad functional group tolerance while revealing that only the *N*-nitro unit on N1 participates in nitroarene formation [8]. According to Yao et al., SO_3_H-functionalized imidazole ionic liquids (e.g., [MIMBs]HSO_4_, 98.1% yield) enabled efficient *m*-xylene nitration with mitigated process hazards through the evaluation of thermal safety parameters and DFT calculations revealed catalytic and solvent effects of ionic liquids in nitration [9].

In addition, researchers have focused on improving nitration processes through process intensification strategies by integrating advanced technologies including ultrasonication, microwave irradiation, and microreaction technology into conventional nitration frameworks. Nikseresht et al. developed a novel heterogeneous heteropoly acid catalyst (PMA@MIL-53(Fe)), enabled efficient regioselective nitration of phenols under ultrasonic irradiation, achieving 54.98% *o*- and 45.01% *p*-nitrophenol yields within 15 minutes [10]. Umrigar et al. demonstrated a microwave-assisted nitration of monochlorobenzene (MCB) for the efficient synthesis of 2,4-dinitrochlorobenzene (2,4-DNCB), achieving reduced reaction time, minimized waste acid, and enhanced yield/selectivity through optimized parameters (microwave energy, temperature, feed composition) [11].

Recent years have witnessed growing adoption of the continuous-flow technology in nitration processes across laboratory and industrial scales, driven by the reaction's classification as a highly hazardous chemical process [12–15]. Extensive case studies in the literature reveal that flow chemistry offers notable advantages over traditional batch methods. The intrinsic safety advantage of flow systems stems from the dramatically smaller reaction volume and larger specific surface area compared to batch reactors. For intensely exothermic nitration reactions with rapid kinetics, this technology enables uniform mixing, efficient heat removal, and precise control of critical parameters including reaction temperature and reactant stoichiometry. Crucially, continuous operation permits scalable production of nitration compounds while inherently mitigating process hazards through intrinsic safety mechanisms. It is critical to clarify that although continuous-flow nitration processes have been extensively documented for a long time, the flow-chemistry technology discussed here primarily addresses small and exquisite continuous-flow devices (e.g., tubular reactor, chip reactor, small-scale CSTR), which demonstrate distinct advantages in enhanced heat/mass transfer capabilities and inherent safety features compared to conventional configurations. Kulkarni’s review highlights how microreactor-enabled continuous-flow nitration innovatively reimagines conventional methods for managing exothermic and selectivity-sensitive reactions, systematically analyzing four distinct approaches (nitration with mixed acids; nitration with fuming nitric acid; vapor-phase nitration; nitration with solid acid catalysts) to assess their advantages, limitations, and scalability potential in miniaturized systems [16]. However, a large number of literature cases on continuous-flow nitration reactions has emerged over the past decade, and there is a lot of general content that can be summarized from the research on continuous-flow nitration processes. Such intensified efforts align with the growing awareness that the inherent risks of nitration reactions – fueled by thermal runaway incidents in batch reactors – have driven the shift toward safer continuous-flow technologies. While systematically reviewing recent advancements in flow-chemistry applications for nitration processes, this work establishes a practical framework to guide researchers in developing continuous-flow nitration systems. The analysis facilitates rapid understanding of fundamental principles and operational mechanisms governing continuous nitration reactions through structured knowledge integration.

## Review

### Flow nitration process

#### Design of continuous-flow nitration system

**Figure 2:**
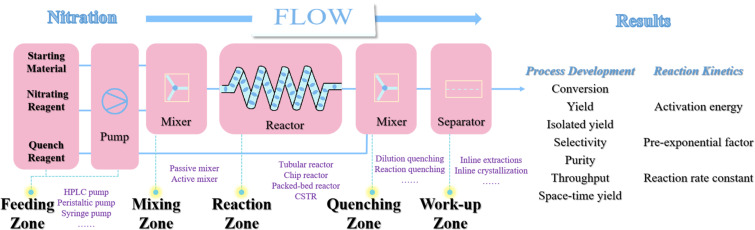
A typical continuous-flow nitration reaction system.

Extensive literature documents various continuous-flow nitration methodologies, with a substantial portion demonstrating transferable operational parameters. Figure 2 presents a schematic diagram of a generic continuous-flow system for nitration reactions. Continuous-flow nitration systems are typically divided into several regions: the feed zone, the mixing zone, the reaction zone, the quenching zone, and the work-up zone.

**Figure 3:**
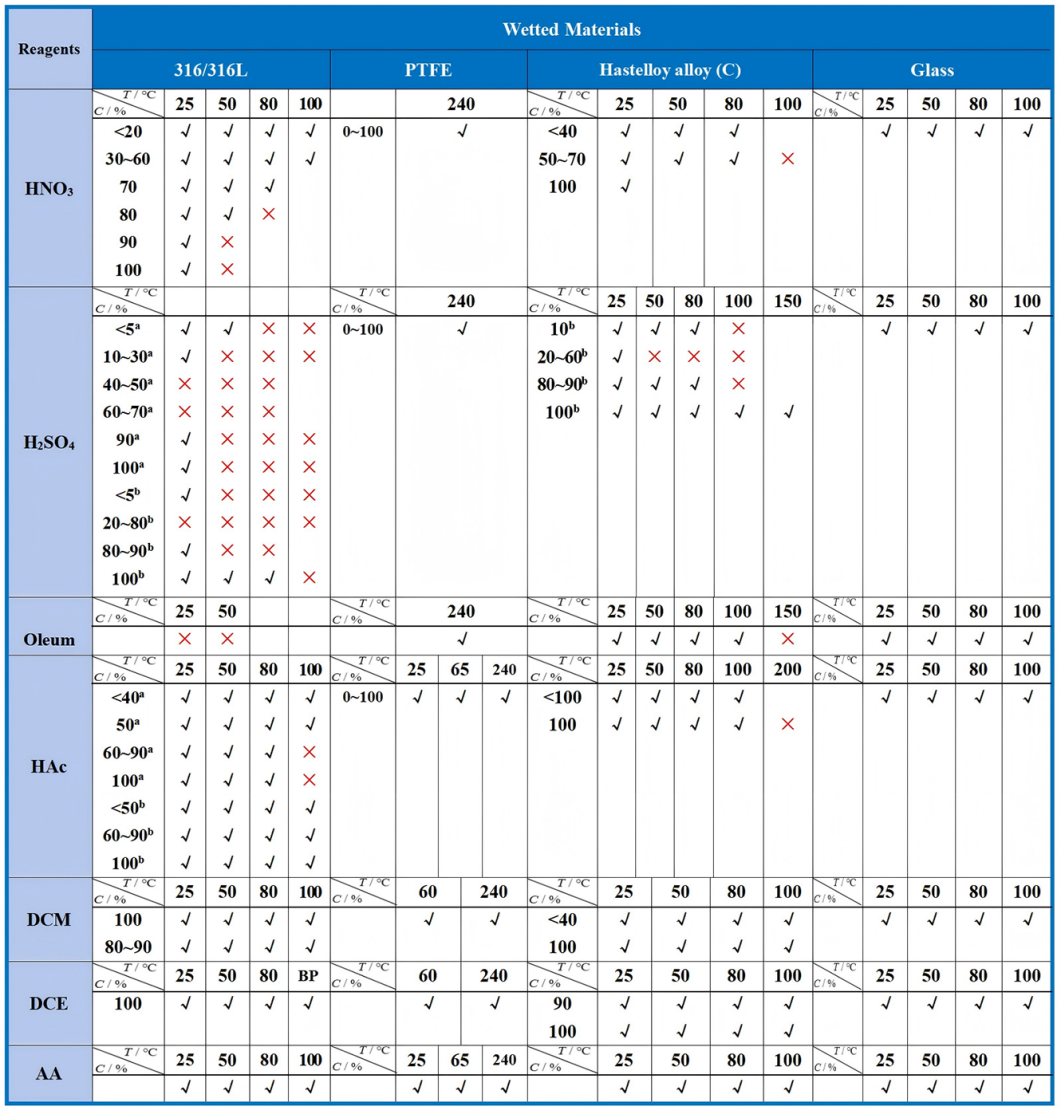
Corrosion characteristics of common wetted materials used in continuous-flow nitration system. Notes: ^a^aerated; ^b^de-aerated. HAc: acetic acid; DCM: dichloromethane; DCE: dichloroethane; AA: acetic anhydride.

When constructing a continuous-flow nitration system, the first consideration is the selection of equipment materials to cope with the corrosiveness of various chemical reagents involved in the nitration process. Common chemical reagents encountered in nitration reactions include nitric acid, sulfuric acid, fuming nitric acid, acetic acid, etc. The materials for equipment in contact with these reagents typically include 316L stainless steel, PTFE, Hastelloy^®^, etc. Figure 3 lists the corrosion characteristics of common wetted materials used in continuous-flow nitration systems [17]. As indicated in Figure 3, operating at reduced temperatures is critical for 316L stainless steel reactors processing concentrated H_2_SO_4_ to minimize corrosion risks. Besides, the selection of materials for the nitration system equipment not only needs to consider the initial state of these reagents but also their changes during the reaction process. For example, 316L stainless steel demonstrates corrosion resistance in individual exposures to concentrated nitric acid or sulfuric acid due to passivation layer formation. However, during nitration using both acids simultaneously, progressive HNO_3_ consumption and water generation may reduce acid concentrations below critical passivation thresholds, necessitating rigorous evaluation of 316L material's corrosion susceptibility under such dynamic operating conditions.

The **feeding zone** of a continuous-flow nitration system typically comprises precision fluid-handling equipment including HPLC pumps, peristaltic pumps, or syringe pumps. The selection criteria must account for operational pressure thresholds, reagent rheological properties (e.g., viscosity), and required flow regimes. A representative challenge arises when handling high-viscosity nitration agents like the HNO_3_/H_2_SO_4_ binary acid system for which HPLC pumps frequently exhibit significant flow rate deviations between programmed and actual delivery rates under high-flow conditions. This flow instability can be mitigated through a strategic thermal management of reagents (30–60 °C) or implementing back-pressure regulation (0.5–2 bar) via inert gas pressurization in storage vessels. In mixed acid nitration systems, the typical liquid–liquid biphasic nature critically governs reaction efficiency through interfacial mass transfer dynamics. Continuous-flow configurations provide diverse mixing solutions ranging from passive geometries (T-junction, Y-junction, heart-shaped designs) to active mixers with various operational modes. Strategically, the **mixing zone** can be positioned upstream of the reaction channel or seamlessly integrated within the reaction module itself to optimize multiphase contact duration and intensity. Fast and strong exothermic reactions are important characteristics of many nitration reactions. The **reaction zone** of a continuous-flow nitration system usually includes four types of reactors: tubular reactor, chip reactor, packed-bed reactor, and CSTR unit. The reactor selection protocol is critically determined by the reaction behavior including but not limited to exothermicity profiles, intrinsic reaction rates, and phase behavior. The **quenching zone** of a continuous-flow nitration system can achieve termination of the nitration reaction through dilution, reaction, and other methods. Without a reasonable quenching method, it is difficult to achieve precise control of residence time, which is a key advantage of flow-chemistry technology. Conventional mixed-acid nitration systems utilize aqueous streams to rapidly deactivate nitration activity, necessitating simultaneous heat extraction to mitigate the substantial exothermic energy generated during the dilution process. While many nitration processes usually exhibit rapid kinetics, specific transformations like partial dinitration reactions demonstrate notably slower reaction rates, substantially compromising process throughput. Although thermal activation via temperature elevation (per Arrhenius kinetics) enhances reaction rates, practical implementation remains constrained by critical temperature thresholds dictated by reactant/solvent system volatility and thermal decomposition limits. For elevated-temperature nitration processes requiring superheated conditions, implementing back-pressure regulation within the continuous-flow reactor system becomes essential to maintain precise pressure control and prevent solvent vaporization. Continuous-flow nitration systems can be augmented with integrated process modules including in-line separation units (**work-up zone**), real-time analytical monitoring, and auxiliary functional components to achieve higher levels of system integration.

#### Development of continuous-flow nitration processes

The successful application of the flow-chemistry technology to nitration reactions necessitates a systematic approach, beginning with the judicious selection and strategic implementation of appropriate methodologies. This is particularly crucial given that the advantages of flow chemistry are not universally applicable across all nitration reactions. Consequently, the optimization of such processes must be carefully tailored to align with the specific reaction characteristics and production requirements. To address these challenges and facilitate the effective implementation of flow chemistry in nitration processes, we have conducted a comprehensive analysis of existing development methodologies over the past decade, focusing on their adaptability and optimization potential within flow-chemistry systems.

The common approaches to developing flow nitration reaction processes generally start with a comprehensive literature review and initial batch reaction studies. These investigations aim to understand the characteristics of the selected nitration reaction, including the reaction rate, the extent of heat generation and the phase behavior during the reaction. During the development of a continuous-flow nitration process, the process parameters to be investigated and their study ranges are determined according to the above-mentioned results. A comprehensive analysis of 39 published articles (2014–2024), selected for their relevance to flow chemistry and nitration process development, is detailed in Table 1 and Table S1 in Supporting Information File 1 [18–56]. This systematic review encompasses critical aspects including operational parameter ranges, nitrating reagents, process analysis methodologies, and reactor configurations. The compiled data and analysis provide valuable insights and guidance for the development of continuous-flow nitration processes, offering researchers a consolidated reference for process optimization and system design.

**Table 1:** Developed continuous-flow nitration processes over the past decade.^a^

Sources	Reaction scheme			
	scope of process parameters research	nitration substrates/ nitrating reagents	analysis methods and results	reactor forms

Wang et al. (2024) [18]	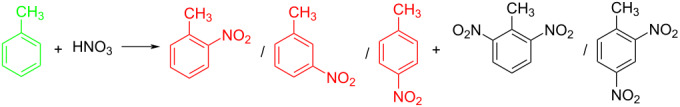			

	solvent-free *T* = 0–60 °C *M-*ratio (N/SM) = 1.0–2.0 *w*_AA_ (%) = 0–80% *t* = 60–180 s	aromatic compounds/ HNO_3_–Ac_2_O	DoE solvent-free *T* = 30 °C *M*-ratio (N/SM) = 1.5 *w*_AA_ (%) = 65% *t* = 120 s yield = 99.21%	316L SS tubular reactor (ID, 0.8 mm; OD, 1.6 mm)

Cao et al. (2024) [19]	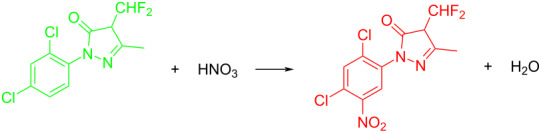			

	1,2-dichloroethane (DCE) SM/DCE = 1:5 (w/w) *M-*ratio (N/S) = 1/8–1/8 *M-*ratio (N/SM) = 1.05–2.0 *T* = 40–70 °C *t* = 25.7–90.0 s	aromatic compounds/fuming HNO_3_–fuming H_2_SO_4_	OFAT solvent DCE SM/DCE = 1:5 (w/w) *M-ratio* (N/S) = 1/6 *M-ratio* (N/SM) = 1.1 *T* = 60 °C *t* = 30 s yield = 97%	Hastelloy C276 chip microreactor (a three-layer structure of 2 heat transfer layers and a reaction layer)

Raimondi et al. (2015) [55]	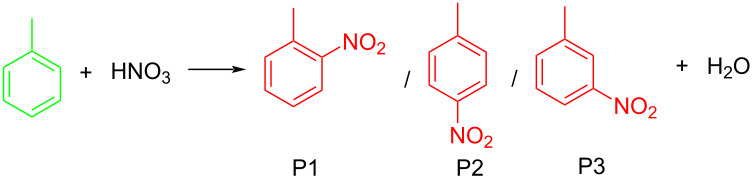			

	solvent-free acid strength = 0.75–0.80 *T* = 23–35 °C *t* = 40–50 s *Q*_SM_ = 0.9–1.1 L/h *Q*_MA_ = 1.4–1.8 L/h *M-*ratio (SM/HNO_3_) = 1.5	aromatic compounds/ HNO_3_–H_2_SO_4_	OFAT solvent-free acid strength = 0.80 *T* = 27 °C *t* = 50 s *M-ratio* (SM/HNO_3_) = 1.5 conv. 33.7% sel. = 95.1% P1:P2:P3 = 54.8/40.5/4.6	SiC heat exchanger chip reactor (square section meandering channel, 2 mm in depth)

Tibhe et al. (2014) [56]	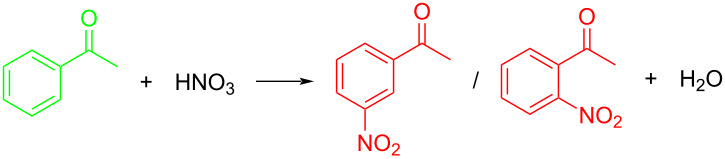			

	H_2_SO_4_ *T* = 0–25 °C *t* = 3–10 min SM/H_2_SO_4_ (*Q*_SM_) = 1:2.58 (v/v) HNO_3_/H_2_SO_4_ (*Q*_MA_) = 1:1.5 (v/v) *Q*_SM_/*Q*_MA_ = 0.8–2 v/v	aromatic compounds/ HNO_3_–H_2_SO_4_	OFAT solvent H_2_SO_4_ *T* = 10 °C *t* = 10 min SM/H_2_SO_4_ (*Q*_SM_) = 1:2.5 (w/v) HNO_3_/H_2_SO_4_ (*Q*_MA_) = 1:1.5 (v/v) *Q*_SM_/*Q*_MA_ = 1:1.66 (v/v) conv. = 100% yield = 98.55%	316L SS tubular reactor

^a^Table S1 in Supporting Information File 1 contains the comprehensive version of Table 1; *M-*ratio (N/SM): the molar ratio of HNO_3_ to starting material, *w*_AA_: the mass fraction of acetic anhydride, w/w: weight/weight, *M*-ratio (N/S): the molar ratio of HNO_3_ to H_2_SO_4_, *Q*_SM_: the flow rate of SM, *Q*_MA_: the flow rate of mixed acid).

Applications of flow chemistry in nitration reactions have predominantly focused on aromatic compounds (89% in Table S1 in Supporting Information File 1, vs 11% for aliphatic counterparts) due to their predictable electrophilic substitution mechanisms and relatively mild reaction conditions, which enable superior controllability and regioselectivity. Unlike aromatic nitrations that predominantly follow ionic mechanisms, aliphatic systems governed by free radical-mediated pathways require significantly more stringent reaction parameters and meticulous acid composition control. These systems frequently exhibit competing reaction channels that typically result in complex mixtures of isomeric by-products, presenting substantial challenges in achieving both regioselectivity and product purity.

**Figure 4:**
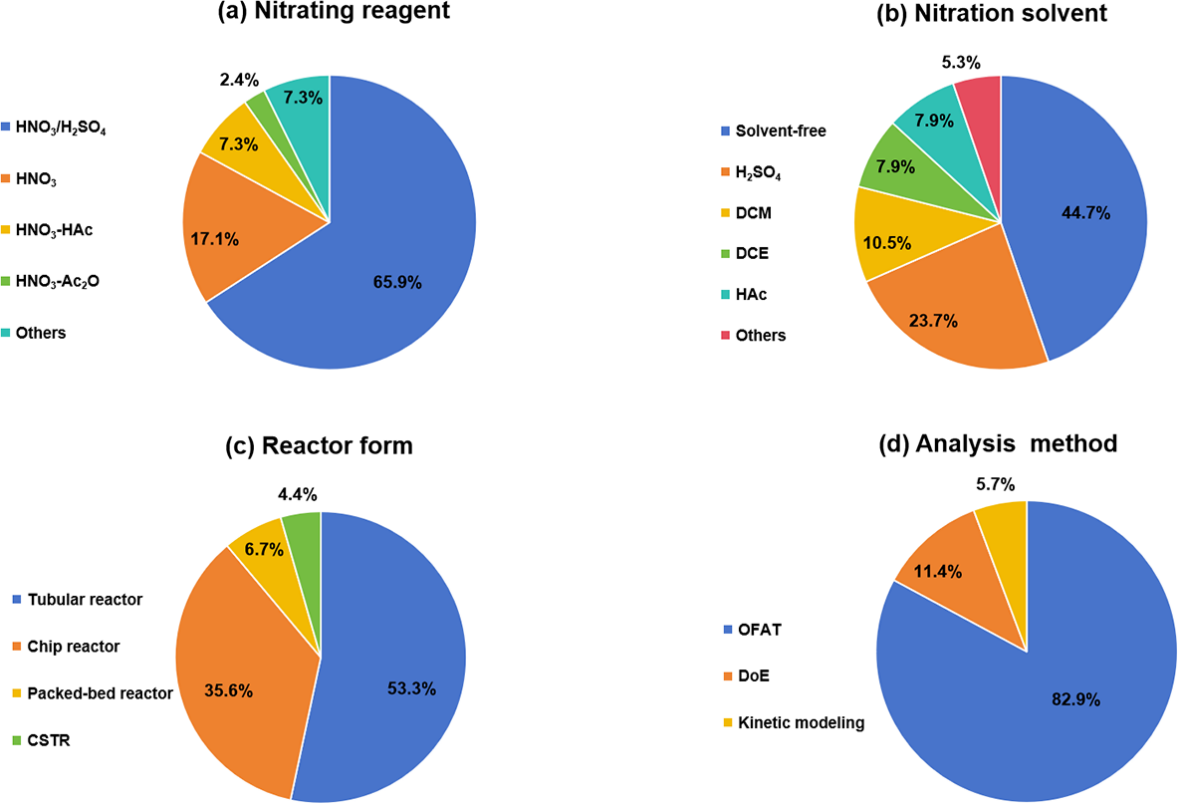
Analysis of the literature on continuous-flow nitration reaction over the past decade.

**Nitrating reagents:** Nitrating reagents play a pivotal role in determining the efficiency, selectivity, and safety of nitration processes, with their selection being fundamentally dependent on the nature of the substrate and desired reaction outcomes. Analytical data (Figure 4a) indicate that 83% of continuous-flow nitration systems employ conventional nitric acid (HNO_3_) or mixed acids of HNO_3_/H_2_SO_4_ as primary nitrating agents. The enduring prevalence of conventional nitrating reagents, especially the mixed acid system (HNO_3_–H_2_SO_4_), in continuous-flow operations stems from their cost-efficiency, predictable reaction profiles, and extensive industrial validation through decades of application. Modern innovations in nitration chemistry have introduced alternative reagents such as dinitrogen pentoxide (N_2_O_5_), particularly in applications requiring enhanced environmental compliance or specific product distribution patterns.

**Nitration solvents:** The selection of solvents for nitration reactions significantly influences reaction kinetics, selectivity, and process safety, requiring careful consideration of both chemical compatibility and operational parameters. Traditional batch nitration processes usually necessitate substantial solvent usage to ensure adequate thermal control and hazard mitigation. In contrast, flow-chemistry systems, leveraging their intrinsic process safety and enhanced mass/heat transfer efficiency, achieve solvent-free conditions in 45% of continuous-flow nitration systems (Figure 4b). A significant proportion of nitration substrates are in the solid state, necessitating solvent-mediated dissolution to achieve practical operability in continuous-flow configurations. The common solvents used in nitration reactions include sulfuric acid (24%), dichloromethane (11%), dichloroethane (8%). and acetic acid (8%) (Figure 4b), which are particularly valued for their ability to dissolve both organic substrates while maintaining chemical stability under the reaction conditions. Notably, sulfuric acid plays a dual role – as catalyst and solvent – in these systems, a multifunctionality stemming not only from its strong proton-donating capacity but also its unique capability to stabilize reactive intermediates through preferential solvation effects. The optimal solvent choice must balance multiple factors: substrate solubility, nitrating reagent compatibility, temperature control requirements, and environmental considerations, with modern trends emphasizing the development of sustainable, recyclable solvent systems that minimize waste generation and energy consumption.

**Reactor forms:** In continuous-flow nitration applications, tubular reactors (53%) and chip reactors (36%) are dominating reactor configurations due to their alignment with the technical demands of nitration processes (Figure 4c). The widespread adoption of tubular reactors stems not only from their superior mass transfer efficiency, plug-flow characteristics, and precise temperature control for highly exothermic reactions, but more significantly from their remarkable cost advantages. Chip-based reactors, offer exceptional heat and mass transfer capabilities due to their high surface-to-volume ratios, ensuring precise temperature control and enhanced safety, particularly for highly exothermic nitration processes. Packed-bed reactors (7%) and continuous stirred-tank reactors (CSTRs, 4%) are less prevalent, as their applications are often limited to specific scenarios – packed beds for catalytic nitration or multi-phase reactions. While small-scale chip and tubular reactors face inherent plugging risks in reactions involving solids and precipitations, CSTRs provide robust solids-handling solutions through controlled suspension mechanisms. The selection of reactor configurations for continuous-flow nitration systems is dictated by an interplay of technical and engineering criteria, encompassing reaction kinetics profiles, heat transfer characteristics, inherent safety protocols, and economic scalability parameters.

**Process parameters:** The implementation of continuous-flow nitration processes rely on the precise control of multiple interdependent parameters, including but not limited to residence time, reaction temperature, nitrating reagents selection, molar ratio of nitric acid to substrate, molar ratio of nitric acid to sulfuric acid, water content, as systematically discussed in Table 1 (vide infra). The residence time (usually 0–120 s) determines reaction completion, requiring balance between conversion rate, throughput, and side reactions. The reaction temperature (usually 0–60 °C) governs kinetic rates and selectivity, where lower temperatures suppress polynitration reactions while higher temperatures demand enhanced heat dissipation. The molar ratio of HNO_3_/substrate (usually 1.0–3.0) directly regulates the nitration degree, with excess nitric acid driving reactivity at the cost of post-treatment complexity. Simultaneously, the molar ratio of H_2_SO_4_/HNO_3_ (usually 1.0–4.0) modulates acid strength and catalytic activity, necessitating effective balancing to optimize nitronium iongeneration efficiency while suppressing byproduct formation from over-sulfonation and minimizing environmental burdens through reduced waste acid discharge. Water content governs the formation equilibrium of nitronium ions (NO_2_^+^), the electrophilic nitrating species, thereby dictating the overall reaction rates. In nitration processes, particularly within heterogeneous systems, mixing efficiency serves as a critical operational parameter. This can be effectively achieved through reactor channel structural design or flow rate adjustments, which ensure effective mass/heat transfer to prevent localized overheating and by-product formation.

**Analysis methods:** A statistical analysis (Figure 4d) indicates that over 80% of analyses for continuous-flow nitration processes employ the one-factor-at-a-time (OFAT) methodology [57]. This approach inherently relies on scientific intuition, where experimental campaigns are conducted through iterative parameter modification, by systematically varying an individual parameter while maintaining others constant. The prevalence of OFAT in the analysis of continuous-flow nitration processes stems principally from its operational simplicity and it does not require advanced mathematical-modeling expertise. In continuous-flow nitration processes, critical process parameters exhibit inherent interdependencies rather than operating as isolated variables. Design of experiments (DoE) represents a systematic statistical approach that constructs mathematical relationships between reaction inputs (e.g., temperature, residence time, stoichiometric ratios) and process outputs (e.g., yield, selectivity, impurity profiles) [57] and merely 11.4% of analysis studies employ DoE methods. While statistically robust within defined operational boundaries, these data-driven models fundamentally differ from mechanistic interpretations – they establish empirical correlations devoid of embedded physicochemical principles. Consequently, DoE-derived predictions maintain validity strictly within the experimentally investigated parameter space, with extrapolation beyond these boundaries remaining inherently speculative. This phenomenological nature limits their utility for fundamental process understanding despite providing practical optimization guidance under constrained conditions. The Kulkarni group [35–36] established a kinetics model for the nitration reaction in synthesizing the selective herbicide pendimethalin using exclusively nitric acid under continuous-flow conditions, aiming to optimize process parameters. The distinctive feature of this kinetics modeling approach lies in its mechanistic foundation rather than statistical correlation. Mechanistic models are constructed based on scientific understanding of chemical processes, as opposed to statistical models that rely solely on empirical relationships between experimental factors and outcomes. While kinetics modeling enables extrapolation of reaction predictions beyond experimentally tested conditions, it demands substantial expertise in reaction mechanism elucidation and presents significant mathematical complexity when dealing with intricate reaction systems.

**Long-term operation stability/stress testing:** Ensuring long-term operational stability is a critical requirement for the practical implementation of continuous-flow nitration processes, particularly given the highly exothermic nature and safety sensitivity of nitration reactions. Unlike batch systems, continuous reactors demand sustained performance under steady-state conditions, necessitating rigorous evaluation of material compatibility and system robustness – a process that becomes even more crucial in nitration systems where minor deviations can lead to thermal runaway or hazardous by-product accumulation. Therefore, conducting long-term operational stability testing becomes imperative following the development of optimized continuous-flow nitration processes to validate industrial viability and ensure reliable performance under extended production cycles, as these tests can reveal progressive channel fouling and blockage or reactor corrosion issues specific to nitration chemistry. Stress testing protocols are systematically designed to probe process boundaries by intentionally exceeding nominal operating parameters (e.g., elevated temperatures approaching the decomposition threshold of nitro compounds, prolonged residence times that may promote polynitration, or extreme reagent stoichiometries that could generate unstable intermediates) while monitoring key performance indicators such as conversion efficiency, selectivity drift toward undesirable polynitrated derivatives, and by-product formation kinetics that are particularly critical in nitration processes. The integration of long-term stability evaluations and stress testing significantly mitigates scale-up failure risks by identifying latent failure modes (e.g., reactor fouling due to precipitation of polynitro by-products or acid-catalyzed polymer formation) that conventional short-term experiments often overlook but are especially prevalent in continuous nitration systems. This systematic validation approach enables predictive maintenance strategies and information about robust reactor design, ultimately bridging the reliability gap between laboratory prototypes and industrial production systems for this particularly challenging class of reactions.

#### Study on nitration reaction kinetics

Although numerous organic nitrating reagents have been developed for nitration reactions, industrial-scale nitration still predominantly relies on inorganic nitrating agents. This is because inorganic reagents (e.g., concentrated nitric acid or mixed acids) offer advantages such as low cost, well-established and stable reaction conditions, scalability for mass production, and broad substrate applicability. Nitration reactions using inorganic nitrating reagents predominantly occur through two distinct mechanistic pathways: free radical and ionic mechanisms. The free radical-mediated nitration pathways using inorganic nitrating reagents remain less explored, with the mechanism predominantly operating in gas-phase nitration systems using aliphatic substrates [2]. In contrast, the ionic mechanism has been extensively investigated, particularly in aromatic nitration systems where it constitutes the predominant pathway. However, these mechanistic classifications are not absolute – an aliphatic nitration may occasionally proceed via ionic pathways, whereas certain aromatic systems demonstrate free radical-mediated reactivity. A notable example of free radical involvement in aromatic nitration was reported by Murray et al., who elucidated a •NO_2_ radical substitution mechanism during the HNO_3_-mediated nitration of arylboronic acids, providing mechanistic justification for this synthetically valuable transformation [58]. Parallel evidence came from Plouffe and colleagues microreactor studies on salicylic acid nitration, where kinetics analysis revealed an autocatalytic profile at 45 °C characterized by a distinct 50-second induction period preceding measurable conversion [59]. The ionic mechanism, particularly well-established for aromatic substrates, involves the acid-catalyzed generation of nitronium ion (NO_2_^+^) through nitric acid dehydration. This electrophilic species subsequently engages in an aromatic electrophilic substitution, with reaction kinetics heavily influenced by the electronic nature of substituents and acid strength. Table 2 analyzes studies in the literature that utilize the flow-chemistry technology to investigate the kinetics of nitration reactions over the past decade.

**Table 2:** Studies on reaction kinetics of continuous-flow nitration reactions over the past decade.

Source	Substrate	Main product	Nitrating reagent	Phase behavior	Kinetics analysis

Cao et al. (2024) [19]	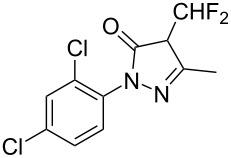	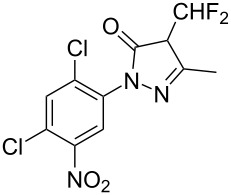	HNO_3_–H_2_SO_4_	liquid–liquid two phase (solvent DCE)	second-order reaction; *E*_a_ = 40.204 kJ/mol lnA = 21.97
Guo et al. (2023) [22]	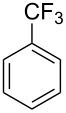	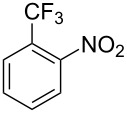 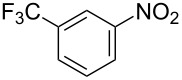 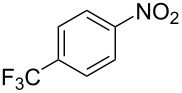	HNO_3_–H_2_SO_4_	homogeneous (solvent H_2_SO_4_)	second-order reaction; *E*_a_ = 86.33 kJ/mol lnA = 47.46 (intrinsic kinetics)
Guo et al. (2023) [23]	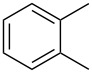	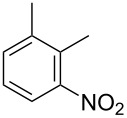 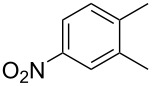	HNO_3_	liquid–liquid two phase (solvent-free)	first-order reaction (large excess of nitric acid); *E*_a_ = 53.05 kJ/mol A = 1.94 × 10^7^ L·mol^−1^·s^−1^
Guo et al. (2023) [24]	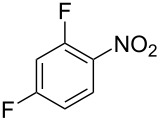	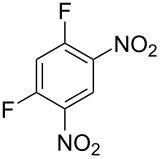	HNO_3_–H_2_SO_4_	homogeneous (solvent H_2_SO_4_)	second-order reaction; *E*_a_ = 60.44 kJ/mol lnA = 39.14 (intrinsic kinetics)
Song et al. (2022) [60]	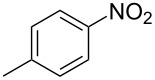	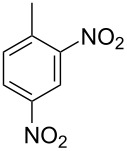	HNO_3_–H_2_SO_4_	homogeneous (solvent H_2_SO_4_)	second-order reaction; *E*_a_ = 50.21 kJ/mol lnA = 58.39 (intrinsic kinetics)
Mule et al. (2022) [30]	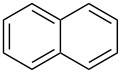	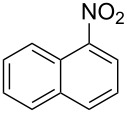 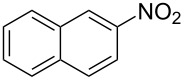	HNO_3_	liquid–liquid two phase (organic solvent)	second-order reaction; for 1-nitronaphthalene A = 2.5232 × 10^5^ *E*_a_/R = 2271.71 for 2-nitronaphthalene A = 1.0067 × 10^5^ *E*_a_/R = 2298.75
Lan and Lu (2021) [32]	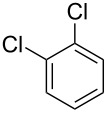	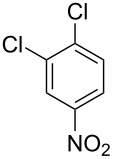 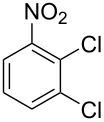	HNO_3_-H_2_SO_4_	liquid–liquid two phase (solvent-free)	second-order reaction; *E*_a_ = 30.96 ± 0.87 kJ/mol
Cui et al. (2022) [61]	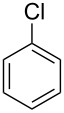	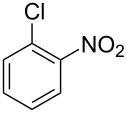 *o*-NCB 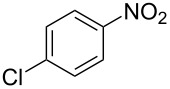 *p*-NCB	HNO_3_–H_2_SO_4_	homogeneous (solvent H_2_SO_4_)	second-order reaction; for *o*-NCB *E*_a_ = 35.12 kJ/mol for *p*-NCB *E*_a_ = 25.92 kJ/mol (intrinsic kinetics)
Kulkarni et al. (2019, 2021) [35–36]	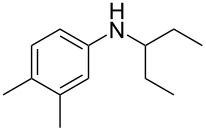	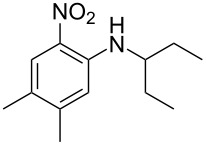 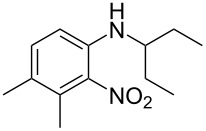 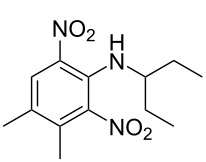 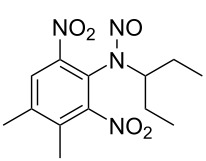	HNO_3_	liquid–liquid two phase (solvent DCE)	second-order reaction [35] *T* = 30/40/50 °C *K*_1_ = 1.7/2.4/4.8 × 10^−6^ L·mol^−1^·s^−1^ *K*_2_ = 0.77/1.6/2.7 × 10^−6^ L·mol^−1^·s^−1^ *K*_3_ = 0.17/0.39/1.7 × 10^−7^ L·mol^−1^·s^−1^
Wen et al. (2018) [67]	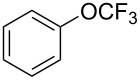	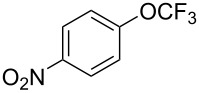 *p*-NB 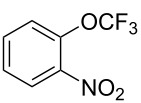 *o*-NB	HNO_3_–H_2_SO_4_	liquid–liquid two phase (solvent-free)	second-order reaction; for *o*-NB *E*_a_ = 29.3 kJ/mol A = 6.3 m^3^·mol^−1^·s^−1^ for *p*-NB *E*_a_ = 26.9 kJ/mol A = 15.4 m^3^·mol^−1^·s^−1^ (intrinsic kinetics)
Li et al. (2017) [50]	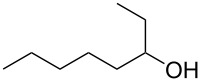	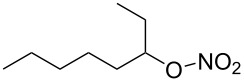	HNO_3_–H_2_SO_4_	quasi- homogeneous (solvent-free)	*E*_a_ = 42.67 kJ/mol A = 6.06 (intrinsic kinetics)
Zhang et al. (2016) [53]	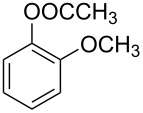	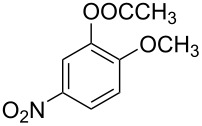 5-NAG 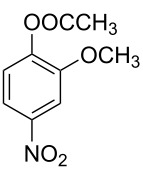 4-NAG	HNO_3_	homogeneous (solvent HAc)	first-order reaction (large excess of nitric acid); For 5-NAG and 4-NAG A = 6.6 × 10^18^ m^3^ mol^−1^·s^−1^) *E*_a_ = 139 kJ/mol For 5-NAG A = 2 × 10^18^ m^3^ mol^−1^ s^−1^ *E*_a_ = 139 kJ/mol For 4-NAG A = 1.15 × 10^12^ m^3^ mol^−1^ s^−1^ *E*_a_ = 101 kJ/mol

Studies on nitration reaction kinetics are predominantly based on the nitronium ion mechanism, with most investigated systems conforming to second-order reaction kinetics. Studies by Guo et al. [23] and Zhang et al. [53] have demonstrated that aromatic nitration reactions follow first-order reactions due to the excessive use of nitric acid, which essentially still adhere to second-order reaction mechanisms.

In kinetics studies of conventional nitration systems, while these processes usually involve liquid–liquid heterogeneous systems, researchers often homogenize the reaction system (e.g., by employing excess sulfuric acid as a solvent) to facilitate kinetics investigations [22,24,50,53,60–61]. This operational strategy is necessitated by the fundamental challenge of decoupling mass transfer effects from intrinsic chemical kinetics in multiphase systems. A well-established framework has emerged for modeling nitration kinetics in homogeneous systems, with the following generalized methodology currently adopted across mechanistic studies.

**Scheme 1:**
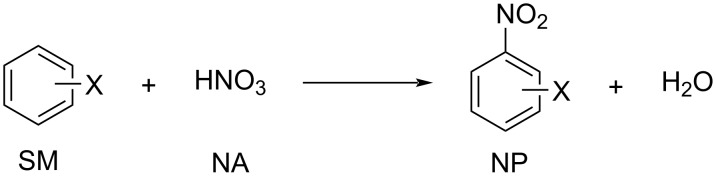
Model reaction for the homogeneous nitration by nitric acid/mixed acid.

**Reaction kinetics in homogeneous systems:** The model reaction for the homogeneous nitration reaction by nitric acid/mixed acid is presented in Scheme 1. It has been proven that a homogeneous nitration reaction is a second-order reaction. Table 3 establishes a fundamental kinetics framework for quantifying this reaction. The **apparent kinetics model** centers on the observed second-order rate constant *k*_obs_ derived from HNO_3_ and SM concentrations, capturing reaction progress through substrate conversion (*X*) and stoichiometric feed ratio (*M*). The linearized integrated form for kinetics analysis can be obtained from the conversion-based rate equation. Therefore, a plot of


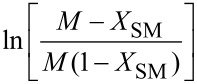


versus *t* is a straight line, and the slope equals 
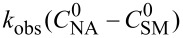
. Next, the observed rate constant based on SM and HNO_3_, *k*_obs_, can be calculated from the slope. Then, the apparent activation energy *E*_a_ for the nitration between HNO_3_ with SM molecules and the pre-exponential factor A can be obtained according to the Arrhenius equation. For example, Zhang et al. [53] investigated the apparent kinetics for the nitration of acetyl guaiacol with fuming nitric acid as the nitrating reagent, in which acetic acid served as solvent to form a homogeneous nitration system.

**Table 3:** Kinetics models for homogeneous SM nitration reactions.

Model	Name	Equation	Description

apparent kinetics model based on HNO_3_ and SM	overall rate equation	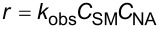	*k*_obs_: observed reaction rate constant based on *C*_NA_ and *C*_SM_ *C*_SM_: the concentrations of SM *C*_NA_: the concentrations of NA
	conversion-based rate equation	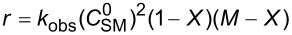 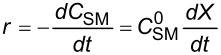 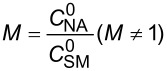	*X*: the conversion of SM 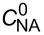 : initial concentrations of HNO_3_ 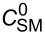 : initial concentrations of SM
	integrated form	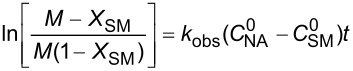	linearized form for kinetics analysis
intrinsic kinetics model based on NO_2_^+^	overall rate equation	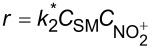	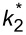 :observed reaction rate constant related to 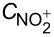 and *C*_SM_ 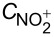 : the concentration of NO_2_^+^
	activity coefficients-based rate equation	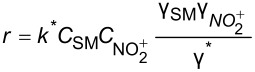	k*: the intrinsic reaction rate constant (solely determined by reaction temperature and unaffected by sulfuric acid concentration) γ_SM_: activity coefficients of SM 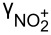 : activity coefficients of NO_2_^+^ γ*: activity coefficients of the transition intermediate
	*M*_c_ function-correlated rate equation	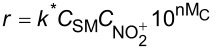 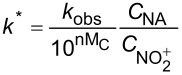	*M*_c_ function: describe the relation between activity coefficient and acidity *n*: a thermodynamic parameter (depends on the nitration substrate)
	integrated form	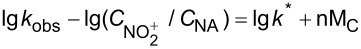	linearized form for kinetics analysis
	model validation	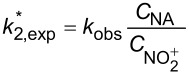 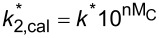	the agreement between calculated and experimental values of 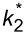 provides critical validation for the kinetics model.

In the continuous-flow nitration system with the nitration reagents of mixed acid, H_2_SO_4_ is a solvent and source of the active electrophilic NO_2_^+^ species. The attack of NO_2_^+^ on the nitration substrates is the rate-determining step in the nitration reaction. Therefore, the effect of sulfuric acid concentration on the nitration reaction must be considered to obtain intrinsic kinetic data, which is based on NO_2_^+^. The **intrinsic kinetics model** based on NO_2_^+^ can be established as shown in Table 3. According to the Brønsted–Bjerrum rate law (transition-state theory) [62], the overall rate equation based on NO_2_^+^ can be transferred into an activity coefficients-based rate equation. According to the literature reported by Marziano et al. [63], the introduction of the *M*_c_ function allows the activity coefficients-based rate equation to be reformulated as *M*_c_ function-correlated rate equation, explicitly linking sulfuric acid acidity to reaction rates. Therefore, the linearized integrated form for kinetics analysis can be obtained from the *M*_c_ function-correlated rate equation. The linearized equation requires prior determination of two key parameters: (i) the acidity function *M*_c_ and (ii) the concentration ratio of 
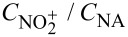
, to resolve the values of k^*^ and *n*. According to the works of Marziano et al. [64–66], quantitative correlations between the *M*_c_ function and both sulfuric acid concentration and temperature could be established. The values of 
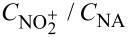
 are governed by both temperature and sulfuric acid concentration. The experimental determination of 
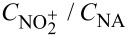
 across varying temperature and H_2_SO_4_ concentrations has been widely reported in the literature. For example, Song et al. [60] proposed a mathematical model to obtain reliable values for 
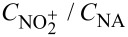
 according to the results reported in the previous literature. The thermodynamic parameters *n* and k^*^ are resolved from the slopes and intercepts, respectively, of linear regression fitted to plots of 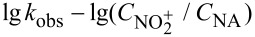 versus *M*_c_ at multiple temperatures. Beyond validating kinetics models through direct comparison of predicted and experimental reaction results under varied conditions, agreement between calculated and experimental rate constants (*k*_2_^*^) provides another verification of model validity. Using the Arrhenius equation, the activation energy *E*_a_ for NO_2_^+^ attack on SM molecules and the pre-exponential factor A can be determined from the values of k^*^ obtained at multiple temperatures. The researches for the mixed acid nitration in homogeneous systems usually conducted the kinetics study to achieve apparent reaction kinetic rate constant and intrinsic kinetic parameters [22,24,50,60–61].

**Reaction kinetics in liquid–liquid biphasic systems:** The kinetics analysis of nitration reactions in liquid–liquid biphasic systems presents unique challenges due to interfacial mass transfer limitations coupled with complex reaction networks. Usually, the kinetics investigation of nitration reactions in liquid–liquid biphasic systems can be categorized into three distinct classes:

**Type A** (**lumped kinetics**): The determination of lumped kinetic parameters is based on HNO_3_ and the mass transfer behavior is coupled with the reaction kinetics. The lumped kinetics of the nitration of naphthalene were determined by Mule et al. [30] and used for modeling CSTR operation. Hussain et al. [35] and Kulkarni et al. [36] determined the lumped kinetics for the dinitration of *N*-(1-ethylpropyl)-3,4-dimethylaniline using nitric acid and the model was formulated to predict the reaction outcome at large capacity. Lan and Lu [32] developed a method for the rapid determination of lumped kinetics for the nitration of *o*-dichlorobenzene by processing the profile of adiabatic temperature rise in a micropacked-bed reactor.

**Type B** (**apparent kinetics**): The determination of apparent kinetic parameters is based on HNO_3_ and the mass transfer behavior is explicitly decoupled from the reaction kinetics. Cao et al. [19] conducted the apparent kinetics study on the nitration of 2-(2,4-dichlorophenyl)-4-(difluoromethyl)-5-methyl-1,2,4-triazol-3-one in a continuous-flow microreactor, in which the effect of mass transfer was ignored due to the significantly enhanced mass transfer efficiency by microreaction technology. Second Damköhler number (Da_II_) is often used to express the time-scale ratio of the mass transfer rate to the chemical reaction rate. Guo et al. [23] established a kinetics model for the nitration of *o*-xylene to determine the apparent kinetics and calculated the value of Da_II_ in the kinetics experiments (<<1) in which the nitration reaction in the microreactor was proved to be kinetically controlled.

**Type C** (**intrinsic kinetics**): The determination of intrinsic kinetic parameters is based on NO_2_^+^ and the mass transfer behavior is explicitly decoupled from the reaction kinetics. The Hatta number (Ha) is a dimensionless quantity used to characterize the relative rates of a chemical reaction and mass transfer in biphasic reaction systems. Wen et al. [67] investigated the intrinsic kinetics for the nitration of trifluoromethoxybenzene with a mixed acid in microchannel reactors, in which the Hatta numbers were calculated to be all smaller than 0.1, confirming the nitration reaction took place in the slow regime and was kinetically controlled.

In nitration reaction kinetics studies across liquid–liquid biphasic systems (types A–C), the complexity of kinetics studies increases progressively from type A to type C, while their applicability expands correspondingly. Kinetics models derived from type A systems are typically confined to the specific experimental conditions under which they were developed, exhibiting limited extrapolation potential beyond those parameters. In contrast, type C kinetics models demonstrate robust extrapolation capabilities, enabling reliable predictions across a broader range of experimental conditions beyond the original study scope. Therefore, the selection of an appropriate kinetics model for liquid–liquid biphasic nitration systems requires a careful balance between the effort invested in model development and the breadth of application scenarios demanded by practical needs.

#### Practical application of continuous-flow nitration

The continuous-flow nitration technology, characterized by its enhanced mass/heat transfer efficiency, precise reaction control, and inherent safety, has emerged as an important alternative to conventional batch-mode nitration processes. Although laboratory-scale continuous-flow nitration has exhibited notable benefits, overcoming the challenges of last mile in transitioning these optimized processes to industrial-scale implementation is essential for practical adoption. Scale-up of nitration processes encompasses two critical dimensions: systematic considerations (e.g., safety) and fundamental challenges (e.g., mass/heat transfer limitations).

**Process safety assessment:** Nitration reactions pose significant safety risks due to their highly exothermic nature (Δ*H* ≈ −73 to −253 kJ/mol) [35], which can trigger thermal runaway if heat release is uncontrolled, and the inherent chemical hazards associated with nitro group (-NO₂) incorporation – a structural hallmark of explosive compounds. The reaction system typically contains excess nitrating agents (e.g., HNO_3_) and generates energetic products, with risks further amplified by the formation of polynitrated by-products (e.g., dinitro/trinitro derivatives). These hazardous by-products arise from the tendency of electron-rich substrates to undergo overnitration, drastically increasing detonation potential. Consequently, rigorous safety assessments are critically important for processes involving nitration reactions.

**Figure 5:**
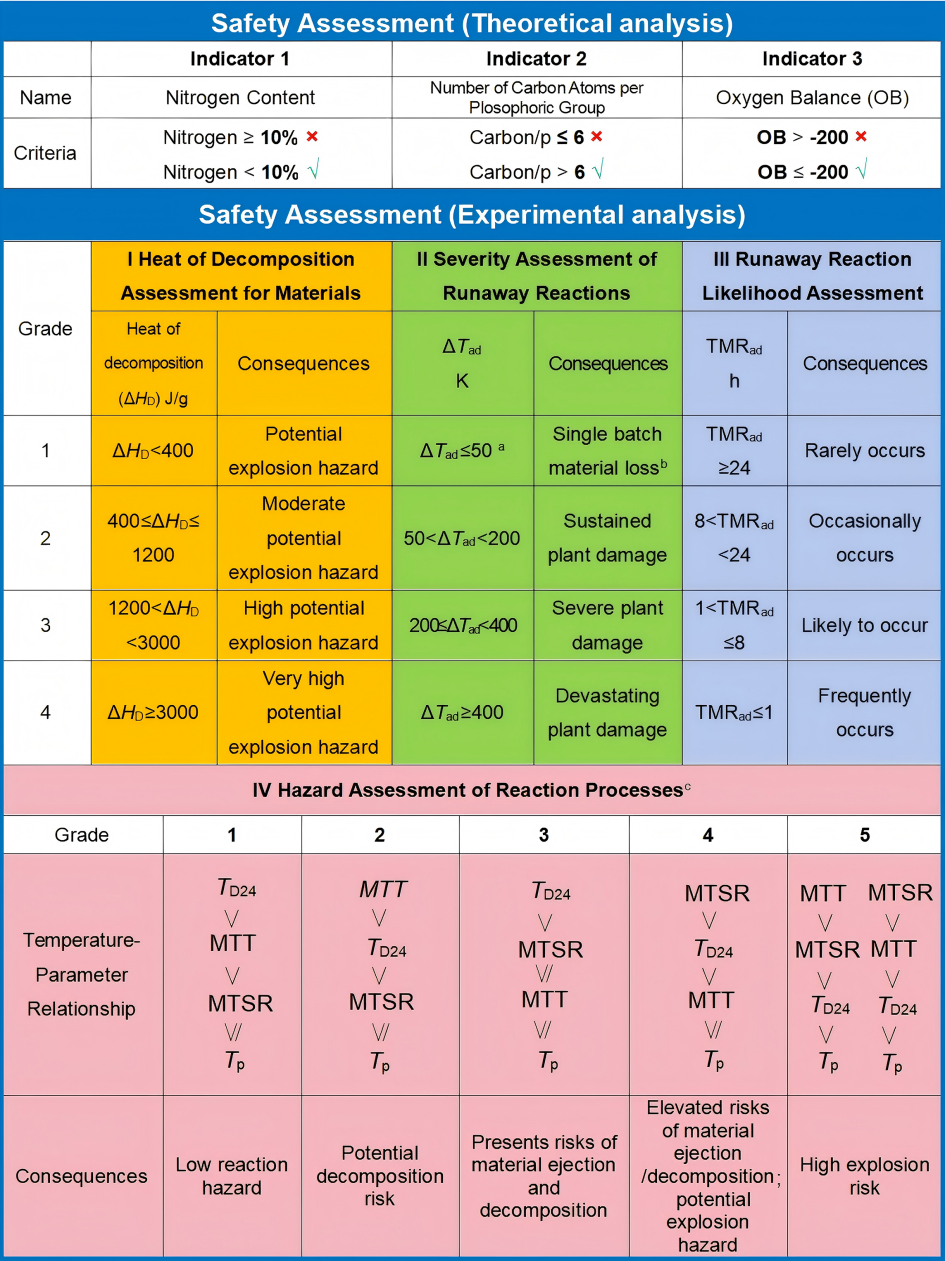
Safety assessment criteria for nitration reactions. Notes: ^a^pressure-independent; ^b^no hazards arising from gas-induced pressure buildup; ^c^*T*_D24_ can be determined by DSC/C80/ARC, MTSR can be determined by RC1, MTT and *T*_P_ are determined by process condition; Δ*H*_D_: heat of decomposition; Δ*T*_ad_: adiabatic temperature rise; TMR_ad_: time to maximum rate under adiabatic conditions; *T*_D24_: the initial temperature when TMR_ad_ equals 24 h; MTT: maximum temperature for technical reasons; MTSR: maximum temperature of the synthesis reaction; *T*_P_: temperature of process.

The safety of nitration reaction processes can initially be theoretically assessed based on the following three indicators (Figure 5) to evaluate the inherent hazards of the involved chemical substances:

Indicator 1: **nitrogen content** [68]. Organic materials with a total nitrogen content below 10 wt % are generally considered non-explosive; it is noteworthy that organic peroxides constitute a notable exception to this rule and require stringent safety measures.

Indicator 2: **number of carbon atoms per plosophoric group** [69]. Materials with nitro functional groups may be classified as plosophoric/explosophores. While a single plosophoric group does not inherently confer explosivity, it suggests thermal instability potential. However, structural dilution (>6 carbons per plosophoric group) typically mitigates explosivity, though thermal instability risks may persist.

Indicator 3: **oxygen balance (OB) of the material** [70]. Oxygen balance (OB), a critical parameter quantifying how well an explosive provides its own oxidant, reveals potential safety risks when OB values exceed the threshold of −200. Since the energy release predominantly originates from oxidation reactions, the available oxygen content critically dictates explosive performance. The oxygen balance for a chemical reaction is calculated by:


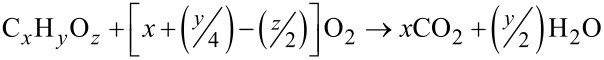


using the formula:


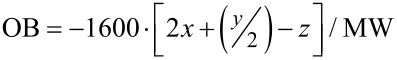


The development needs of industrial nitration processes have been for many years addressed using batch processes, in which the corresponding equipment is easily assembled and cleaned, and their performances have long been established. Safety considerations are often a critical driver for transitioning nitration processes from traditional batch methods to continuous systems, thus necessitating a comprehensive safety assessment of conventional batch operations. When assessing the safety risks associated with traditional batch nitrations, the following criteria (Figure 5) could be applied [71]:

I Evaluate the explosive hazard of materials based on their heat of decomposition.

II Evaluate the severity of runaway reactions based on adiabatic temperature rise.

III Evaluate the likelihood of runaway reactions based on the time to maximum reaction rate.

IV Evaluate the hazard level of reaction processes based on relevant temperature parameters.

For more detailed assessment criteria, please consult the corresponding literature [71]. The hazard level of traditional batch nitration processes should be comprehensively evaluated against the four aforementioned criteria. For synthesis processes assessed as high-risk, adopting flow chemistry technologies is strongly recommended to mitigate reaction hazards and operational risks. Wang et al. [18] examined the nitration reaction calorimetry in a nitric acid/acetic anhydride system under semi-batch conditions, revealing an average reaction enthalpy of −173.63 kJ·mol^−1^ – significantly more exothermic than conventional nitration processes, with this enhanced exothermicity likely arising from concurrent heat release during acetic anhydride hydrolysis. Chen et al. [38] revealed that the batch nitration of 3-fluorobenzotrifluoride with fuming nitric acid poses significant thermal hazards through the reaction calorimeter (RC1e) test as rapid temperature surges during the initial stage of the nitric acid addition and post-reaction DSC analysis confirmed exothermic activity of the unquenched organic phase containing products, emphasizing persistent risks even after the unquenched post-nitration system. Rakshit et al. [47] conducted the synthesis of 6-nitrovanillin through continuous-flow nitration using fuming HNO_3_ in sulfolane efficiently and safely, in which ARSST, DSC, and RC1e were utilized to study the specific safety hazards of this reaction.

Existing safety assessments of nitration reactions in the literature above primarily focus on conventional batch-reactor systems, while emerging continuous-flow processes lack comprehensive safety assessment. However, such assessment is critically needed, as the transition from batch to continuous-flow operation often entails significant modifications to critical process parameters and may even involve fundamental alterations to reaction pathways. Glotz et al. [72] developed a novel continuous-flow setup to measure reaction enthalpies (Δ*H*_r_), determining Δ*H*_r_ for the nitration of phenol nitration as −121 kJ/mol and providing key safety assessment data for this hazardous reaction. Here, a relatively simple method can be utilized for process-safety assessment of the developed continuous-flow nitration process due to the inherent safety features (particularly in microreactor systems). In addition to evaluating it based on the three parameters mentioned above (the number of carbon atoms per plosophoric group, oxygen balance (OB) of the material, and nitrogen content), the raw materials, products, by-products, and reaction mixtures involved in the continuous-flow nitration process were analyzed by differential scanning calorimetry (DSC) to determine the onset temperature of exothermic reactions. This onset temperature was then reduced by a specified value (recommended: 30–100 K) to establish the safe upper operating temperature limit for the continuous-flow process. For example, Priestley et al. [73] proposed that subtracting 60 K from the onset temperature of the exothermic reaction provides a recommended safe upper temperature limit for laboratory-scale ball milling synthesis operations based on DSC results. Compared to batch-based nitration processes, conducting nitration reactions in continuous-flow systems offers a significantly broader thermal operating window. Studying the exothermic behavior through techniques such as DSC and RC1 helps guide the inherently safe design of continuous-flow nitration scale-up processes. HAZOP (hazard and operability study) also serves as a pivotal safety assessment methodology in continuous-flow nitration process development, leveraging its systematic framework for risk identification and process safety optimization. For instance, the nitration of toluene is employed as a test reaction to identify critical failure scenarios leading to thermal runaway in continuous intensified heat exchanger (HEX) and semi-continuous reactors, as systematically analyzed by Raimondi et al. using the HAZOP methodology [55].

**Scale up:** The scale-up extent of nitration processes is closely tied to the application fields of the resulting nitration compounds, with significant variations in production demands across industries. For high-value, low-volume sectors such as flavors and fragrances and pharmaceuticals, typical production scales fall within tens to hundreds of tons per year. In contrast, bulk chemical industries including agrochemicals, dyes, and fine chemicals often operate at thousands to tens of thousands of tons annually. Nevertheless, many successful applications of flow-chemistry technology, emerged as a pivotal approach in industrial nitration processes, focused on the production of pharmaceutical intermediates, energetic materials, and functional chemical intermediates. These specialized products are characterized by low annual throughput requirements, high market value, and extreme sensitivity to reaction parameters (e.g., thermal runaway risks, competing nitration pathways) – operational challenges that align perfectly with flow chemistry's inherent advantages in process intensification and intrinsic safety. The relatively high costs of flow chemistry-related equipment, the sunk costs of existing batch reactors, the scarcity of skilled flow-chemistry personnel, and the inherent technical challenges collectively limit the widespread application of flow chemistry in the continuous transformation of nitration processes across different industries.

Table 4 demonstrates the recent cases for the scale up of continuous-flow nitration reactions. For scale up of nitration process using continuous-flow, several approaches are listed in the literature, e.g., external numbering up, internal numbering up and sizing up [74]. In the numbering-up approach, ensuring a uniform flow distribution in parallel channels is difficult. The numbering-up approach inherently struggles with uniform flow distribution across parallel microchannels due to manufacturing tolerances, reactor fouling-induced pressure imbalances, and gas bubble entrapment, leading to localized deviations in nitration kinetics and consequent product quality inconsistencies in industrial-scale operations. Despite inherent scaling hurdles in numbering-up continuous-flow nitration reactions – such as flow maldistribution – targeted strategies like dimensionless number correlations (Reynolds, Euler) and multiscale simulation frameworks integrating micro-/macro-effects show promise in bridging the gap between lab-scale feasibility and industrial scalability. Typically, the preferred method to address the scale-up of continuous-flow nitration processes is through sizing up, combined with appropriate structural design to compensate for the attenuation of mass and heat transfer performance caused by the increased dimensions [35–36,39,48]. The adjustment of process operation parameters (e.g., increasing volumetric flow rates) is another method to compensate for attenuated transfer performance [18,21,25]. Although different mass transfer compensation methods can improve transfer characteristics to some extent, achieving full compensation remains highly challenging, particularly for nitration systems that are critically dependent on mass transfer efficiency. For example, in the nitration of naphthalene studied by Xu et al. [21], the yield of the target product decreased from 94.96% to 87.81%, even when increasing volumetric flow rates were employed to compensate for mass transfer attenuation after scale-up. Nevertheless, all scale-up cases summarized in Table 4 achieved lab-scale equivalent performance or industrially acceptable outcomes, thereby highlighting the potential of continuous-flow reactors in bridging laboratory development with industrial-scale manufacturing of hazardous nitration processes.

**Table 4:** Scale up of continuous-flow nitration reaction in recent years.^a^

Source	Nitration substrates	Key process parameters and results		compensation methods

		bench scale	scale-up	

Wang et al. (2024) [18]	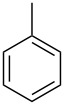	*Q* = 1.5 mL/min *t* = 120 s yield = 99.21% STY = 3.877 g·L^−1^·s^−1^ TP = 1.00 kg/d	*Q* = 18 mL/min *t* = 120 s yield = 98.32% STY = 2.884 g·L^−1^·s^−1^ TP = 8.97 kg/d	increasing volumetric flow rates [316L tubular reactor (ID 0.8 mm, OD 1.6 mm) → 316L tubular reactor (ID 0.8 mm, OD 1.6 mm)]
Xu et al. (2023) [21]	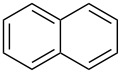	*Q* = 3 mL/min *t* = 120 s *T* = 30 °C H_2_SO_4_ strength = 74% yield = 94.96 %	*Q* = 60 mL/min *t* = 120 s *T* = 30 °C H_2_SO_4_ strength = 74% yield = 87.81% STY = 1.575 g·L^−1^·s^−1^ TP = 21.14 kg/d	increasing volumetric flow rates [Teflon tubular reactor (ID 0.8 mm, OD 1.6 mm) → 316L tubular reactor (ID 4.35 mm, OD 6.35 mm)]
Mittal et al. (2023) [25]	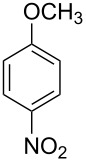	*Q* = 0.8 mL/min *T* = 80 °C *t* = 2.5 min *M*-ratio (N/SM) = 2.5 HPLC purity = 98%	*Q* = 6.4 mL/min *T* = 80 °C *t* = 2.5 min *M*-ratio (N/SM) = 2.5 HPLC purity = 99+% isolated yield = 90% TP = 0.6 kg/d	Increasing volumetric flow rates [PFA tubular reactor (ID 1 mm, OD 1.6 mm) → 316L tubular reactor (ID 4.35 mm, OD 6.35 mm)]
Fu et al. (2022) [28]	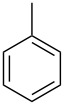	N/A	*Q* = 60 mL/min *T* = 45 °C *t* = 2.2 min *M*-ratio (N/SM) = 1.2 SPY = 1.36 g·L^−1^·s^−1^ TP = 20.6 kg/d	N/A [Hastelloy chip reactor (1/4 inch OD)]
Sagandira et al. (2021) [33]	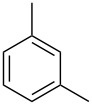	Uniqsis reactor *T* = rt *t* = 6 min *V*-ratio (N/S) = 50:50 yield_total_ = 90% sel. (2,4-nitro) = 95% sel. (2,6-nitro) = 5%	LTF reactor *T* = rt *t* = 6 min *V*-ratio (N/S) = 50:50 yield_total_ = 90% sel. (2,4-nitro) = 95% TP (2,4-nitro) = 0.4 kg/d	N/A [glass chip reactor (Uniqsis, I.V. 2 mL) → chip reactor (Little Things Factory, I.V. 4.5 mL)]
Köckinger et al. (2020) [39]	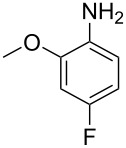	*Q*_SM&Ac2O_ = 1.06 mL/min *Q*_MA_ = 0.049 mL/min *T* = 20 °C/20–30 °C *M*-ratio (N/SM1) = 1.15 *M*-ratio (S/SM1) = 1.15 isolated yield = 82% HPLC purity = 99+% TP = 0.1344 kg/d	*Q*_SM&Ac2O_ = 89.2 mL/min *Q*_MA_ = 3.4 mL/min *T* = 30 °C isolated yield = 83% HPLC purity = 99+% TP = 48 mol/d	specific reactor structural design [Hastelloy C22 chip microreactor & PFA tubular reactor (OD 1/8 inch, ID 0.8 mm) → flow plate A5 rack chip microreactor]
Kulkarni et al. (2021) [35] and (2019) [36]	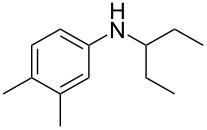	N/A	TP = 50 kg/d	specific reactor structural design [316L tubular reactor (OD 1/8 inch) → pinched tubular reactor (OD 1/4 inch, distance of successive pinchings 3 cm)]
Wen et al. (2017) [48]	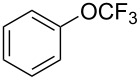	*M*-ratio (N/SM) = 1.1 *M*-ratio (S/N) = 4 water content φ = 97 wt % *T* = 273 K *Q*_or_ = 0.4 mL/min *Q*_aq_ = 0.9 mL/min conv. = 99.6% sel. (*p*/*o*/*m*-nitro) = 90.97:7.26:0.08% sel. (dinitro) = 1.04%	*M*-ratio (N/SM) = 1.02 *M*-ratio (S/N) = 4 *Q*_or_ = 10 mL/min *Q*_aq_ = 24.2 mL/min *t* = 2.4 min *T* = <−2 °C TP = 24 kg/d conv. = 99.6% sel. (*p*/*o*/*m*-nitro) = 91.08:7.05:0.08% sel. (dinitro) = 1.34%	specific reactor structural design [SS tubular reactor (ID 0.6 mm) → 16 channels chip microreactor & tubular-packed reactor (packed with cylindrical quartz sand microparticles)]

^a^*Q*: the flow rate; STY: space time yield; TP: throughput; *V*-ratio (N/S): the volume ratio of HNO_3_/H_2_SO_4_; *M*-ratio (N/SM): the molar ratio of HNO_3_ to SM; *Q*_SM&Ac2O_: the flow rate of SM and Ac_2_O; *Q*_MA_: the flow rate of mixed acid; *M*-ratio (N/SM1): the molar ratio of HNO_3_ to SM1; *M*-ratio (S/SM1): the molar ratio of H_2_SO_4_ to SM1; *M*-ratio (S/N): the molar ratio of H_2_SO_4_ to HNO_3_; *Q*_or_: the flow rate of organic phase; *Q*_aq_: the flow rate of aqueous phase.

**Figure 6:**
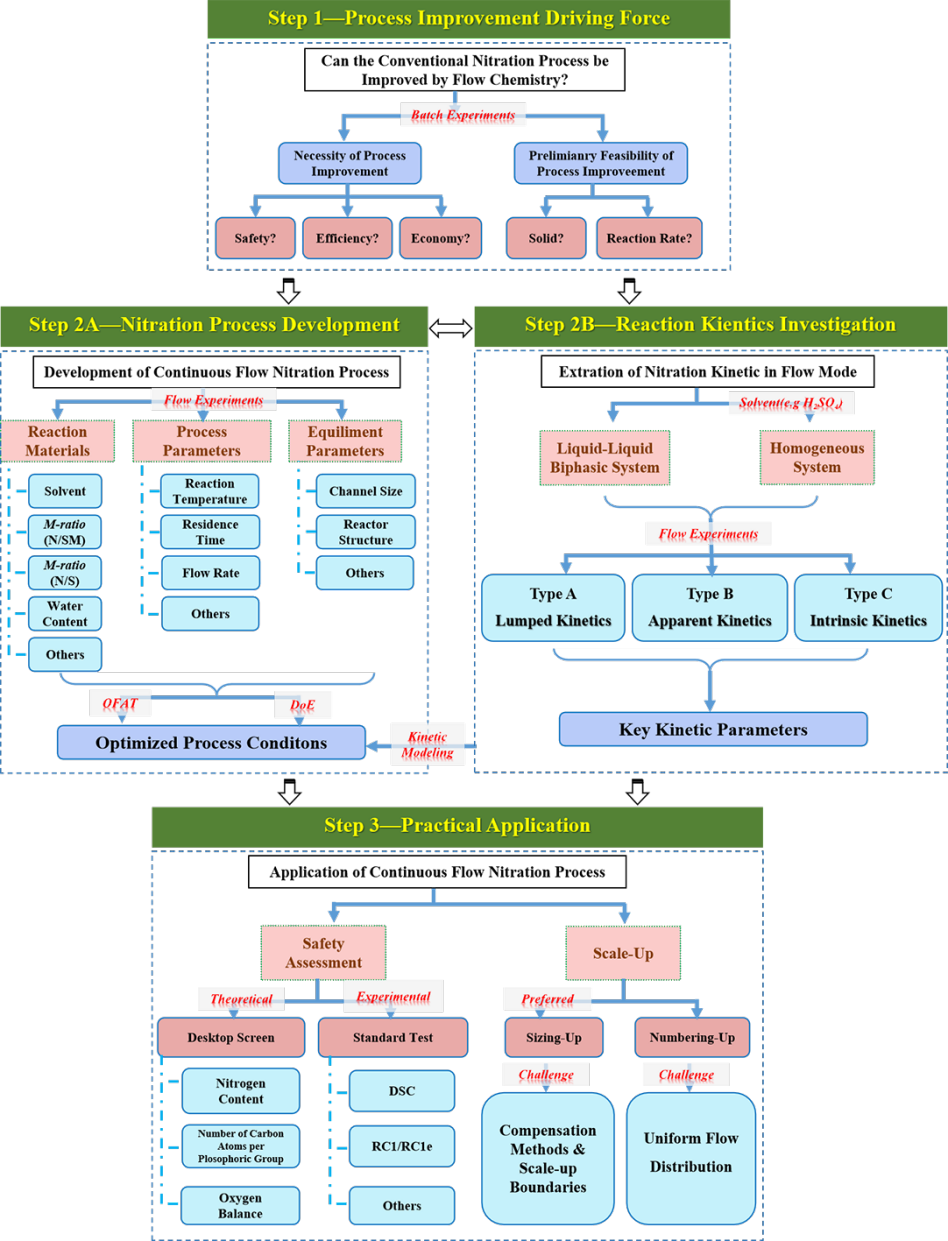
Guide for the investigation of continuous-flow nitration processes.

Nitration processes remain one of the most hazardous chemical processes globally due to their intrinsic risks of explosive decomposition and thermal runaway reactions. Such processes are subject to stringent regulatory oversight under national safety mandates. Flow chemistry demonstrates revolutionary advantages for safer nitration process intensification. Prior to the presentation of definitive conclusions, we anticipate delivering practical strategies to assist researchers in leveraging flow chemistry for the advancement of continuous-flow nitration systems (Figure 6).

The first step is to determine whether conventional nitration processes could benefit from flow chemistry (step 1, Figure 6). This evaluation primarily involves three aspects – safety, efficiency, and economics – to assess the necessity of adopting the continuous-flow technology. Additionally, the selection of a specific continuous-flow nitration approach (tubular/chip/CSTR/packed-bed reactor) depends on two critical factors: the presence of solids in the reaction and the reaction rate. Although CSTRs tolerate moderate solid-phase systems, these parameters remain decisive in determining the technical feasibility of flow-based nitration processes.

The second step in continuous-flow nitration process investigation can proceed via direct nitration process development (step 2A, Figure 6) or nitration reaction kinetics investigation (step 2B, Figure 6). The nitration process exploration (in step 2A) helps define parameter boundaries (e.g., temperature, residence time, reagent ratios) for subsequent kinetics studies, while reaction kinetics analysis (step 2B) provides mechanistic insights and optimization methodologies (e.g., rate constants) to refine continuous-flow operations. These approaches are complementary: process development guides kinetics modeling, and kinetic data facilitates the determination of optimized reaction conditions.

The ultimate objective (step 3, Figure 6) for the investigation of a nitration process is to translate lab-scale continuous-flow nitration processes into industrially viable systems. Safety, a critical driver for transitioning from traditional batch reactors to continuous-flow, also serves as a guiding principle for ensuring seamless implementation of flow-based nitration. Scale-up strategies, on the other hand, dictate the design of reactor systems (e.g., modular units, reactor configure) necessary for robust practical-scale implementation. Together, safety protocols and scale-up methodologies form the cornerstone of successful batch-to-flow technology transfer in nitration chemistry.

## Conclusion

Flow-chemistry technology has achieved significant progress in the development of nitration reaction processes, analysis of nitration reaction kinetics, and intensification of nitration production processes. By leveraging its excellent heat and mass transfer performance and intrinsic safety characteristics, this technology has effectively mitigated the mass transfer limitations and safety risks associated with traditional nitration processes, substantially improving reaction efficiency. Through analysis of the relevant literature over the past decade, this review aims to examine common process parameters and ranges in continuous-flow nitration process development, universal methodologies for continuous-flow nitration kinetics studies, and effective approaches for scaling up continuous-flow nitration processes. The findings are intended to provide guidance for subsequent researchers attempting to develop continuous-flow nitration reaction processes.

Although flow-chemistry technology has been extensively investigated for nitration reactions, several critical aspects still require refinement. Firstly, current research predominantly focuses on the formation mechanisms of target nitro-products and their isomeric by-products, while crucial side reactions such as oxidation and overnitration remain underexplored. A more comprehensive understanding of these competing pathways is essential for enhancing reaction selectivity and process control. Secondly, mechanistic studies of continuous-flow nitration processes using inorganic nitrating reagents have primarily concentrated on ionic reaction pathways, with free radical-mediated mechanisms and their associated kinetics behavior receiving limited attention. This knowledge gap hinders the optimization of reaction systems where free radical intermediates may play significant roles. Thirdly, systematic theoretical frameworks for scale-up of continuous-flow nitration processes remain underdeveloped. While sizing-up strategies are commonly employed in a nitration process, the absence of universal mass transfer compensation methodologies and validated criteria for determining effective scale-up boundaries poses significant challenges. Addressing these challenges will be crucial for advancing continuous-flow nitration toward sustainable and industrially viable processes.

## Supporting Information

File 1 A complete table of developed continuous-flow nitration processes over the past decade (Table S1) and the nomenclature used in this review.

## Data Availability

Data sharing is not applicable as no new data was generated or analyzed in this study.
