# Supplementary material for: Continuous-flow-enabled intensification in nitration processes: a review of technological developments and practical applications over the past decade
Source: Beilstein J Org Chem. 2025 Aug 26;21:1678–99. doi: 10.3762/bjoc.21.132 (PMC12415922; doi:10.3762/bjoc.21.132)
Supplement: File 1 — A complete table of developed continuous-flow nitration processes over the past decade (Table S1) and the nomenclature used in this review. [file Beilstein_J_Org_Chem-21-1678-s001.pdf]

BEILSTEIN JOURNAL OF ORGANIC CHEMISTRY

Supporting Information

for

Continuous-flow-enabled intensification in nitration processes: a review of technological developments and practical applications over the past decade

Feng Zhou, Chuansong Duanmu, Yanxing Li, Jin Li, Haiqing Xu, Pan Wang and Kai Zhu

Beilstein J. Org. Chem. **2025,** *21,* 1678–1699. doi:10.3762/bjoc.21.132

A complete table of developed continuous-flow nitration processes over the past decade (Table S1) and the nomenclature used in this review

License and Terms: This is a supporting information file under the terms of the Creative Commons Attribution License (https://creativecommons.org/licenses/by/4.0). Please note that the reuse, redistribution and reproduction in particular requires that the author(s) and source are credited and that individual graphics may be subject to special legal provisions.
The license is subject to the *Beilstein Journal of Organic Chemistry* terms and conditions: (https://www.beilstein-journals.org/bjoc/terms)

Table S1: Developed continuous-flow nitration processes over the past decade.

Sources	Reaction Scheme			
	Scope of process parameters research	Nitration substrates/ nitrating reagents	Analysis methods and results	Reactor forms
Wang, J. C. et al (2024) [1]	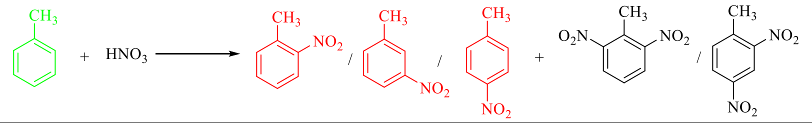			
	**Solvent-free**<br>*T*=0-60 °C<br>*M-ratio*(N/SM)=1.0-2.0<br>w_{AA}(%)=0-80%<br>*t*= 60-180 s	Aromatic compounds<br>/HNO_3-Ac_2O	**DoE**<br>Solvent-free<br>*T*=30 °C<br>*M-ratio*(N/SM)=1.5<br>*w*AA(%)=65%<br>*t*= 120 s<br>**Yield=99.21%**	316L SS tubular reactor<br>(ID, 0.8 mm; OD, 1.6 mm)
Cao, J. Y. et al (2024) [2]	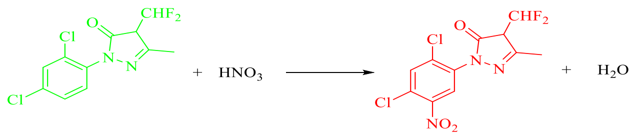			
	**1,2-dichloroethane(DCE)**<br>SM/DCE=1/5 w/w<br>*M-ratio*(N/S)=1/8-1/2<br>*M-ratio*(N/SM)=1.05-2.0<br>*T*=40-70 °C<br>*t*=25.7 s- 90.0 s	Aromatic compounds<br>/Fuming HNO_3-Fuming H_2SO_4	**OFAT**<br>Solvent DCE<br>SM/DCE=1/5 w/w<br>*M-ratio*(N/S)=1/6<br>*M-ratio*(N/SM)=1.1<br>*T*=60 °C<br>*t*=30 s<br>**Yield=97%**	Hastelloy(HC276) chip microreactor (a three-layer structure of 2 heat transfer layers and a reaction layer)
Mittal, A. K. et al (2023) [3]	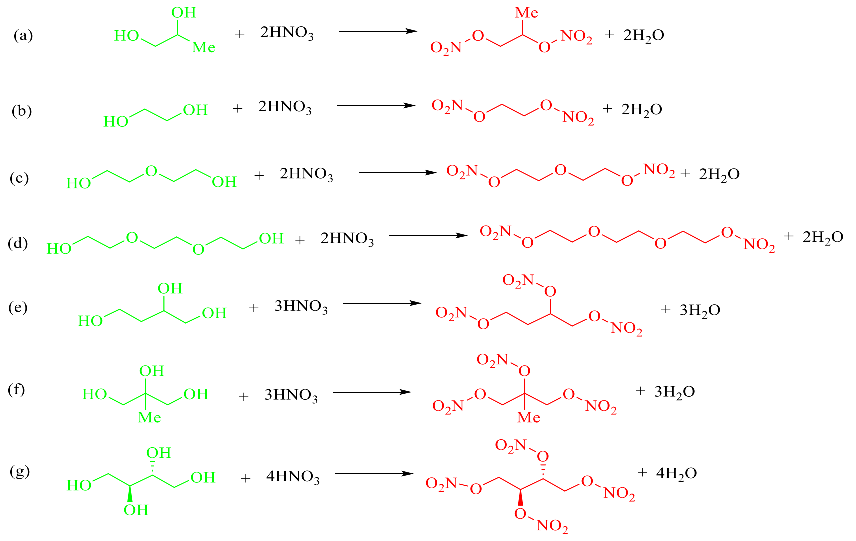			
	**Solvent-free/H_2SO_4**<br>(a) *T*=15 °C, *t*=1-2 min,<br>*M-ratio*(N/SM)=3-5,<br>12.7 M HNO_3<br>(HNO_3/H_2SO_4/H_2O = 51/43/6),<br>Neat SM	Aliphatic compound/HNO_3-H_2SO_4	**OFAT**<br>Solvent-free/H_2SO_4<br>(a)*T*=15 °C, *t*=1 min,<br>*M-ratio*(N/SM)=5<br>12.7 M HNO_3<br>(HNO3/H_2SO_4/H2O=51	PFA tubular reactor (ID, 1 mm; OD, 1/16 inch; IV, 2 mL)

	(b) *T*=20-30 °C, *t*=1-2 min,<br>*M-ratio*(N/SM)=3~4<br>12.7 M<br>HNO_3(HNO_3/H_2SO_4/H_2O =<br>51/43/6), Neat SM<br>(c) *T*=10-40 °C, *t*=1 min,<br>*M-ratio*(N/SM)=3-4<br>12.7 M HNO_3<br>(HNO_3/H_2SO_4/H_2O=51/43/6)<br>Neat SM<br>(d) *T*=10-45 °C, *t*=1 min,<br>*M-ratio*(N/SM)=3-5<br>12.7 M HNO_3<br>(HNO_3/H_2SO_4/H_2O=51/43/6)<br>Neat SM<br>(e) *T*=10 °C, *t*=1-2 min,<br>*M-ratio*(N/SM)=5-6<br>12.7 M HNO_3<br>(HNO_3/H_2SO_4/H_2O=51/45/4)<br>Neat SM<br>(f) *T*=30-80 °C, *t*=1-2 min,<br>*M-ratio*(N/SM)=6<br>2.0 M SM (95% H_2SO_4)<br>Neat HNO_3(95%)<br>(g) *T*=60-80 °C, *t*=1-2 min,<br>*M-ratio*(N/SM)=6-8<br>1.7 M SM (95% H_2SO_4)<br>Neat HNO_3(95%)		/43/6)<br>Neat SM<br>**Yield=93%**<br>(b)*T*=30 °C, *t*=1 min,<br>*M-ratio*(N/SM)=4<br>12.7 M HNO_3<br>(HNO3/H_2SO_4/H2O=51<br>/43/6)<br>Neat SM<br>**Yield=95%**<br>(c)*T*=30 °C, *t*=1 min,<br>*M-ratio*(N/SM)=4<br>12.7 M HNO_3<br>(HNO_3/H_2SO_4/H_2O=51/<br>43/6)<br>Neat SM<br>**Yield=96%**<br>(d)*T*=45 °C, *t*=1 min,<br>*M-ratio*(N/SM)=5<br>12.7 M HNO_3<br>(HNO3/H_2SO_4/H_2O=51<br>/43/6)<br>Neat SM<br>**Yield=95%**<br>(e)*T*=10 °C, *t*=1 min,<br>*M-ratio*(N/SM)=6<br>12.7 M HNO_3<br>(HNO3/H_2SO_4/H_2O=51<br>/45/4)<br>Neat SM<br>**Yield=90%**<br>(f)*T*= 80 °C, *t*=1 min,<br>*M-ratio*(N/SM)=6<br>2.0 M SM (95% H_2SO_4)<br>Neat HNO_3(95%)<br>**Yield=94%**<br>(g)*T*=80 °C, *t*=1 min,<br>*M-ratio*(N/SM)=8<br>1.7 M SM (95% H_2SO_4)<br>Neat HNO_3(95%)<br>**Yield=96%**	
Xu, F. et al (2023) [4]	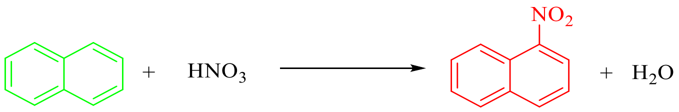			
	**1,2-dichloroethane(DCE)**<br>SM/DCE=1/4 M/M<br>*M-ratio*(N/SM)=0.8-2.0<br>*T*=30-50 °C<br>*t*=10-120 s<br>H_2SO_4 strength=74%-82%	Aromatic compounds<br>/<br>Fuming<br>HNO_3-H_2SO_4	**OFAT**<br>Solvent DCE<br>SM/DCE=1/4 M/M<br>*M-ratio*(N/SM)=1.2<br>*T*=30 °C<br>*t*=120 s<br>H_2SO_4 strength=74%<br>**Yield=94.96%**	Teflon tubular reactor (ID, 0.8 mm; OD, 1.6 mm)
Guo, S. et al (2023) [5]	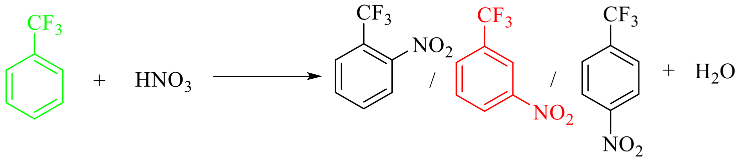			

	**Continuous-flow System A**<br>**Solvent-free**<br>*M-ratio*(N/S)=0.78<br>T=10-20 °C<br>t=0-30 s<br>Conc.(H_2SO_4) =86%-91%<br>*M-ratio*(N/SM)=1.0-1.6<br>**Continuous-flow System B**<br>**Solvent-free**<br>*M-ratio*(N/S)=0.71-0.78<br>*M-ratio*(N/SM)=1.0-1.8<br>T=20-50 °C<br>t=0-30 s<br>Conc.(H_2SO_4) =86%-93%	Aromatic compounds<br>/<br>Fuming HNO_3-H_2SO_4	**OFAT**<br>**Continuous-flow System A**<br>Solvent-free<br>*M-ratio*(N/S)=0.78<br>T=20 °C<br>t=28 s<br>Conc.(H_2SO_4) =91%<br>*M-ratio*(N/SM)=1.2<br>**Conv.=22%**<br>Notes: Not the final optimized result<br>**Continuous-flow System B**<br>Solvent-free<br>*M-ratio*(N/S)=0.71<br>*M-ratio*(N/SM)=1.5<br>t=30 s<br>**Conv.=100%**	**Continuous-flow System A**<br>316L SST T-mixer(ID, 3.3 mm)+PTFE tubular reactor(ID, 1mm)<br>**Continuous-flow System B**<br>Hastelloy(HC276) chip microreactor (a three-layer structure of 2 heat transfer layers and a reaction layer)
Guo, S. et al (2023) [6]	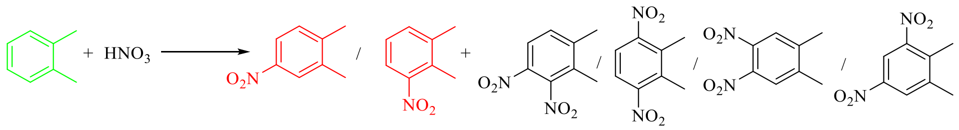			
	**Solvent-free**<br>Q=20-60 mL/min<br>Conc.(HNO_3)=88%-98%<br>*M-ratio*(N/SM)=2-6<br>T=283-323 K<br>t=0-60 s	Aromatic compounds<br>/<br>HNO_3	**OFAT**<br>Solvent-free<br>Q=60 mL/min<br>*Conc.*(HNO_3)=94%<br>*M-ratio*(N/SM)=3.6<br>T=323 K<br>t=9 s<br>**Conv.=100%**	Hastelloy heart-shaped chip microreactor
Guo, S. et al (2023) [7]	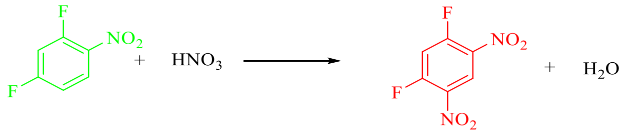			
	**H_2SO_4**<br>**Strategy 1-T-micromixer**<br>*Re*=12.7-45<br>d_i=1.2-1.0 mm<br>T=318-333 K<br>t=0-36 s<br>*M-ratio*(N/SM)=1.1<br>*M-ratio*(S/SM)=6<br>**Strategy 2-Heart-shaped microreactor**<br>*Re*=58-204<br>T=318-333 K<br>t=0-12 s<br>*M-ratio*(N/SM)=1.1<br>*M-ratio*(S/SM)=6<br>w(H_2SO_4)=93%-98%	Aromatic compounds<br>/<br>HNO_3-H_2SO_4	**OFAT**<br>**Strategy 1-T-micromixer**<br>Solvent H_2SO_4<br>T=333 K<br>t=36 s<br>*M-ratio*(N/SM)=1.1<br>*M-ratio*(S/SM)=6<br>**Conv.=35%**<br>**Strategy 2-Heart-shaped microreactor**<br>Solvent H_2SO_4<br>T=333 K<br>t=45 s<br>*M-ratio*(N/SM)=1.1<br>*M-ratio*(S/SM)=6<br>**Conv.=98.5%**	PTFE tubular reactor(ID 1.2 mm/1.6 mm/2 mm)+Hastelloy heart-shaped chip microreactor
Mittal, A. K. al (2023) [8]	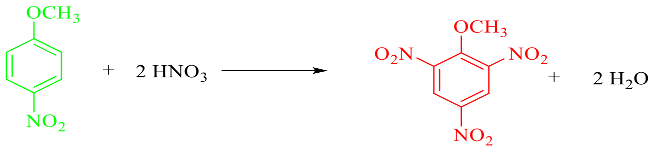			

	**H_2SO_4**<br>Conc.(H_2SO_4-dissolving SM)=95%-98%<br>69% HNO_3 in 98% H_2SO_4<br>*M-ratio*(N/SM)=2.5-3<br>*T*=60-100 ºC<br>*t*=0.5-5 min	Aromatic compounds<br>/<br>HNO_3-H_2SO_4	**OFAT**<br>Solvent H_2SO_4<br>*T*= 80 ºC<br>*t*=2.5 min<br>*M-ratio*(N/SM)=2.5<br>Conc.(H_2SO_4-dissolvin g SM)=98%<br>**HPLC purity=98%**	PFA tubular reactor (ID, 1 mm; OD, 1/16 inch; IV, 2 mL)
Petho, B. et al (2022) [9]	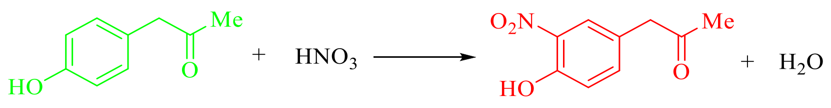			
	**Dichloromethane (DCM) /AcOH**<br>*M-ratio*(N/SM)=1.025-1.1<br>*T*=25-45 ºC<br>*t*=5-60 s<br>*Q*=1-6 mL/min	Aromatic compounds<br>/<br>HNO_3-HAc	**OFAT**<br>Solvent DCM/AcOH<br>*M-ratio*(N/SM)=1.025-1 .1<br>*T*=35-45 ºC<br>*t*=10-60 s<br>*Q*=1-6 mL/min<br>**Conv.(max)=~100%**<br>Note: Parameter adjustment for the actual production needs	Glass chip microreactor
Guo, S. et al (2022) [10]	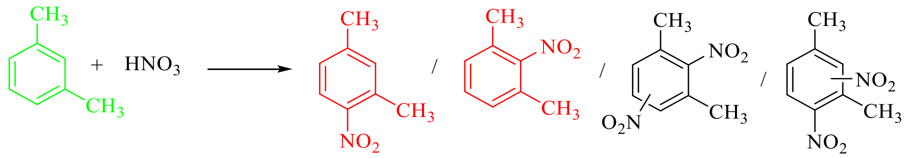			
	**Dichloromethane (DCM)**<br>SM/DCM=1/1 v/v<br>**Stragety 1-Mixed acid**<br>H_2SO_4 content=70-98 wt%<br>HNO_3 content=70-98 wt%<br>*M-ratio*(S/N)=0.8-2.33<br>*M-ratio*(N/SM)=1.2-2<br>*T*=20-50 ºC<br>*t*=1 min<br>**Stragety 2-HNO_3+H_2SO_4**<br>H_2SO_4 content=80-90 wt%<br>HNO3 content=90-98 wt%<br>*M-ratio*(N/SM)=2-3.2<br>*T*=10-40 ºC<br>*M-ratio*(S/SM)=1.5-2<br>*t*=0-120 s	Aromatic compounds<br>/<br>HNO_3-H_2SO_4	**OFAT**<br>Solvent DCM<br>SM/DCM=1/1 v/v<br>**HNO_3+H_2SO_4(two-ste p mononitration)**<br>H_2SO_4 content=80 wt%<br>HNO_3 content=98 wt%<br>*M-ratio*(N/SM)=2.4<br>*T*=40 ºC<br>*M-ratio*(S/SM)=2<br>*t*=104 s<br>**Conv.=99.5%**<br>**Sel.(2,4-nitro)=83.8%**<br>**Sel.(2,6-nitro)=15.2%**	Hastelloy(HC276) chip microreactor(the reaction layer comprises 36 heart-shaped cells)
Fu, G. et al (2022) [11]	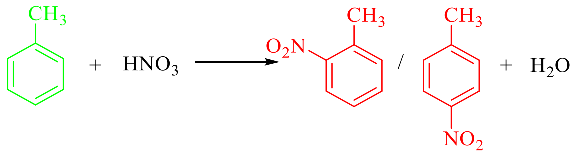			
	**Solvent-free**<br>*Q*=10-60 mL/min<br>*T*=40-60 ºC<br>*t*=2.2-5.1 min<br>*M-ratio*(N/SM)=1-1.4	Aromatic compounds<br>/<br>HNO_3-H_2SO_4	**OFAT**<br>Solvent-free<br>*Q*=60 mL/min<br>*T*=45 ºC<br>*t*=2.2 min<br>*M-ratio*(N/SM)=1.2<br>**SPY=1.36 $g \cdot L^{-1} \cdot s^{-1}$**	Hastelloy chip reactor(the channels were made of 1/4 in o.d hastelloy)

Guo, S. et al (2022) [12]	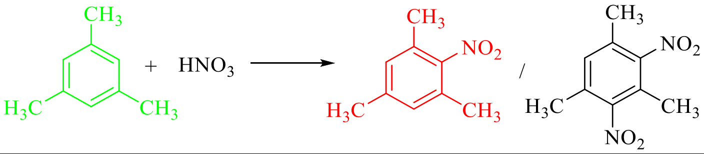			
	**Dichloromethane(DCM)**<br>SM/DCM=1/1 v/v<br>**Stragety 1-Mixed acid**<br>Solvent type=EA/DCM/CS_2 /cyclohexane/None<br>H_2SO_4 content=51-71 wt%<br>*M-ratio*(S/N)=0.6-3<br>*M-ratio*(N/SM)=0-5.9<br>*T*=20-60 °C<br>*Q*=20-70 mL/min<br>*t*=30 s<br>**Stragety 2-HNO_3**<br>*t*=30 s<br>*T*=40 °C<br>*Q*=60 mL/min<br>*M-ratio*(N/SM)=1-4<br>**Stragety 3-HNO_3+Mixed acid**<br>H_2SO_4 content=60-90 wt%<br>*M-ratio*(N/SM)=1.2-3.5<br>*T*=35-45 °C<br>*M-ratio*(S/SM)=0-1.7<br>*t*=0-60 s	Aromatic compounds<br>/<br>HNO_3<br>&HNO_3-H_2SO_4	**OFAT**<br>Solvent DCM<br>SM/DCM=1/1 v/v<br>**Stragety 1-Mixed acid**<br>*M-ratio*(S/N)=0.6<br>*M-ratio*(N/SM)=4.5<br>*T*=60 °C<br>**Yield=94.7%**<br>**Impurtiy=4.4%**<br>**Stragety 2-HNO_3**<br>*M-ratio*(N/SM)=3<br>*T*=40 °C<br>**Yield=93.17%**<br>**Impurtiy=0.8%**<br>**Stragety 3-HNO_3+Mixed acid**<br>*M-ratio*(S/N)=0.6<br>*M-ratio*(N/SM)=2.6<br>*T*=60 °C<br>*t*=60 s<br>**Conv.=99.8%**<br>**Yield=95%**<br>**Purity=97%**<br>**Throughput=1.88 kg/h**	Hastelloy (HC276) chip microreactor(the reaction layer comprises 36 heart-shaped cells)
Mule, G. M. et al (2022) [13]	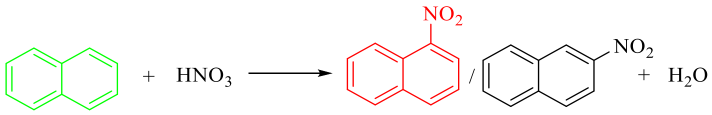			
	N/A	Aromatic compounds<br>/<br>HNO_3	**OFAT**<br>**I** The glass reactor configuration<br>2 CSTRs(1 L) in parallel+1 CSTR(2 L)<br>**Production=148 kg/d**<br>**II** The SS316L reactor configuration<br>2 CSTRs(2 L) in series<br>**Production=218 kg/d**<br>**III** Multipoint dosing<br>3 CSTRs(3 L/5 L/7 L) in series<br>**Production=522 kg/d**	CSTR flow reactor
Sacher, S. et al (2022) [14]	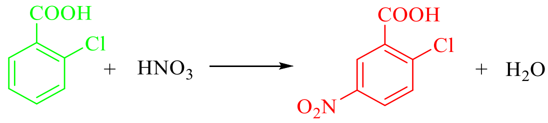			
	**H_2SO_4**<br>Q_{SM}=0.4-1 mL/min<br>Q_A=0.4-1 mL/min<br>T=0-35 °C	Aromatic compounds<br>/<br>HNO_3-H_2SO_4	**DoE**<br>**N/A**	Split and recombine cascade mixer (Cascade Mixer 06)

Lan, Z. et al (2021) [15]	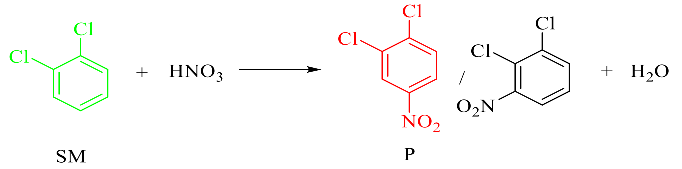			
	**Solvent-free**<br>**ETFE tubular reactor**<br>Conc. (SM, organic phase) =25-100%<br>*T*=10-60 ºC<br>*t*=0-200 s<br>*M-ratio*(N/SM)=0~2<br>*M-ratio*(S/N)=4<br>**Micropacked-bed reactor**<br>Conc. (SM, organic phase) =25-100%<br>*t*=0-36 s<br>*M-ratio*(N/SM)=0~2<br>*M-ratio*(S/(SM+P))=4	Aromatic compounds<br>/<br>HNO_3-H_2SO_4	**OFAT**<br>ETFE tubular reactor<br>Solvent-free<br>*T*=40 ºC<br>*t*=200 s<br>*M-ratio*(N/SM)=1.1<br>*M-ratio*(S/N)=4<br>**Conv.=98.52%**<br>**Sel.=89.02**<br>**STY=0.0693 g/(L·s)**<br>Micropacked-bed reactor<br>Solvent-free<br>*T*=30~70 ºC (Adiabatic)<br>*t*=5 s<br>*M-ratio*(N/SM)=1.1<br>*M-ratio*(S/N)=13.2<br>**Conv.=100%**<br>**Sel.=88.97%**<br>**STY=0.4382 g/(L·s)**	ETFE tubular reactor(ID 0.75 mm, OD 1.60 mm)/316L tubular reactor(ID 4mm, OD 6mm)/316L micropacked-reactor(ID 4mm, OD 6mm, packed with 0.177-0.250 mm/ 0.350-0.500 mm/0.500-0.710 mm glass beads)
Sagandira, M. B. et al (2021) [16]	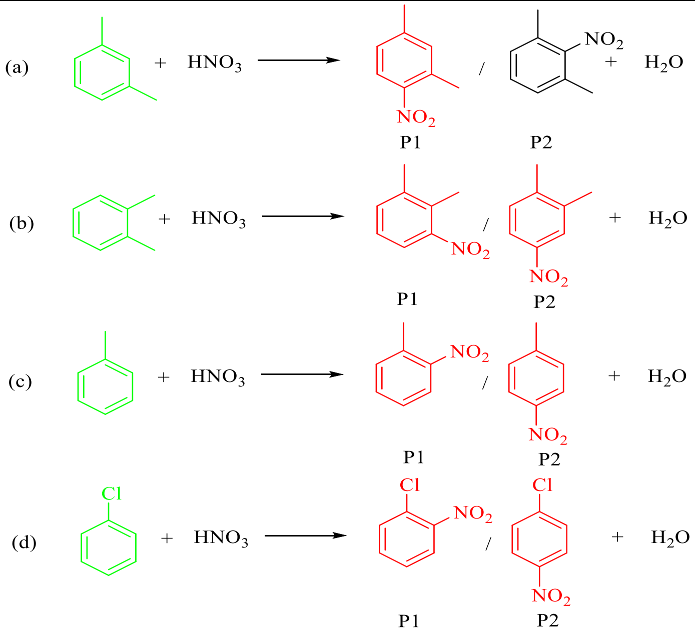			
	Reaction (a)<br>**Solvent-free**<br>**PTFE tubular reactor**<br>Ultrasonic assisted<br>*T*=R.T./60 ºC<br>*t*=0-20 min<br>*V-ratio*(N/S)=10/90-90/10<br>**Uniqsis glass chip microreactor**<br>*T*=R.T.-100 ºC<br>*t*=2-10 min<br>*V-ratio*(N/S)=50/50	Aromatic compounds<br>/<br>HNO_3-H_2SO_4	**OFAT**<br>**(for reaction (a))**<br>**PTFE tubular reactor**<br>Ultrasonic assisted<br>*T*=R.T.<br>*t*=15 min<br>*V-ratio*(N/S)=50/50<br>**(a)**<br>**Yield(P1&P2)=100%**<br>**Sel.(P1/P2)=80%/20%**<br>**(b) Yield(P1&P2)=97%**<br>**Sel.(P1/P2)=58%/42%**<br>**(c) Yield(P1&P2)=97%**<br>**Sel.(P1/P2)=64%/36%**<br>**(d) Yield(P1&P2)=95%**<br>**Sel.(P1/P2)=22%/78%**<br>**Uniqsis glass chip**	Chemtrix glass chip microreactor (300 μm channel width, 120 μm channel depth)/PTFE tubular reactor(ID 0.8 mm)/Uniqsis glass chip reactor

			**microreactor**<br>*T*=R.T.<br>*t*=6 min<br>*V-ratio*(N/S)=50/50<br>**(a) Yield(P1&P2)=90%**<br>**Sel.(P1/P2)=95%/5%**<br>**(b) Yield(P1&P2)=88%**<br>**Sel.(P1/P2)=73%/17%**<br>**(c) Yield(P1&P2)=90%**<br>**Sel.(P1/P2)=75%/25%**<br>**(d) Yield(P1&P2)=90%**<br>**Sel.(P1/P2)=7%/93%**	
Cardinal-David, B. et al (2021) [17]	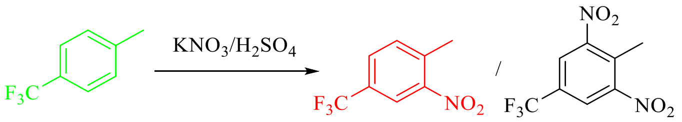			
	**Solvent-free**<br>N/A	Aromatic compounds<br>/<br>KNO_3-H_2SO_4	N/A<br>Solvent-free<br>**Processing amount =50kg SM/24h**<br>**Purity=98+%**<br>**Yield=98+%(potency-adjusted)**<br>**Byproduct= <1%**	CSTR flow reactor
Hussain, A. et al (2021) [18]<br>&<br>Sharma, M. et al (2019) [19]	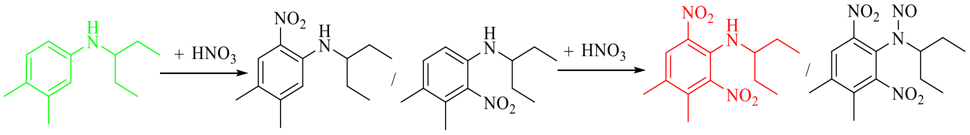			
	**1,2-dichloroethane(DCE)**<br>*t*=7-20 min<br>*T*=50-90 °C<br>*M-ratio*(N/SM)=2.5-3.8<br>Reactant composition=60-90%<br>Notes: Parameters were checked at the pilot scale	Aromatic compounds<br>/<br>HNO_3	**Kinetic Modeling**<br>**Production rate=2 kg/h**	Pinched tubular reactor(D 1/4 inch)
Kyprianou, D. et al (2020) [20]	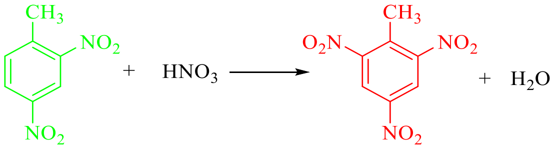			
	**H_2SO_4**<br>Conc.(SM in H_2SO_4)=0.56-1.1 M<br>*T*= 110-150 °C<br>*t*=10-30 min<br>*M-ratio*(N/SM)=1~5	Aromatic compounds<br>/<br>HNO_3-H_2SO_4	**DoE**<br>Conc.(SM in H_2SO_4)=0.56 M<br>*T*= 130 °C<br>*t*=20 min<br>*M-ratio*(N/SM)=3<br>**Conv.=99+%**	Tubular reactor(3 mm diameter)
Chen, P. et al (2020) [21]	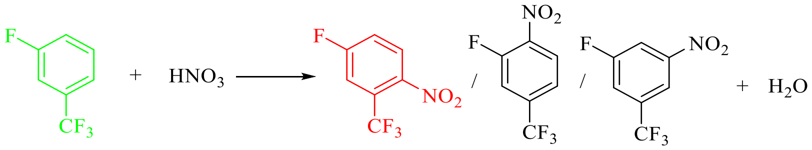			
	**Solvent-free**<br>*T*= -30-0 °C<br>*t*=2-19 min<br>*M-ratio*(N/S)=0.8-9.5<br>*M-ratio*(N/SM)=1.4-4.8	Aromatic compounds<br>/<br>HNO_3-H_2SO_4	**OFAT**<br>Solvent-free<br>*T*= 0 °C<br>*t*=16 min<br>*M-ratio*(N/S)=4.6<br>*M-ratio*(N/SM)=3.77<br>**Yield=96.4%**	Stainless steel tubular reactor(ID 4 mm, OD 6.5 mm)

Köckinger, M. et al (2020) [22]	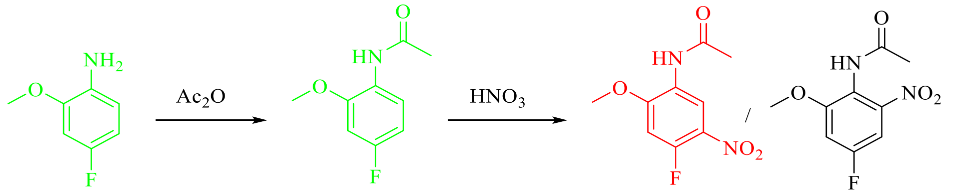<br>SM1 SM2			
	**HAc**<br>(a) Conc.(SM2 in reactor)=0.33/0.5 M<br>*T*=20-80 °C<br>*t*=0.5-16 min<br>*M-ratio*(N/SM2)=15/30<br>(b)Conc.(SM in reactor)=0.364-0.459 M<br>*T*=20/40 °C<br>*t*=40-180 s<br>*M-ratio*(N/SM)=4-16<br>(c)Conc.(SM1 in AcOH)=0.5M<br>*T*=20°C/20-30 °C<br>*M-ratio*(N/SM1)=1.15-3<br>*M-ratio*(S/SM1)=0.15-1.15	Aromatic compounds<br>/<br>(a) 69% HNO_3<br>(b) 100% HNO_3-H_2SO_4<br>(c) 100% HNO_3/fuming H_2SO_4	**OFAT**<br>Solvent HAc<br>(a)Conc.(SM2 in reactor)=0.33 M<br>*T*=80 °C<br>*t*=0.5 min<br>*M-ratio*(N/SM2)=30<br>**Yield=99+%**<br>**Isolated yield=86%**<br>(b) Telescoping of the Acetylation and Nitration<br>Conc.(SM1 in reactor)=0.364 M<br>*T*=20 °C<br>*t*=65 s<br>*M-ratio*(N/SM1)=15<br>**Conv.(SM1)=96%**<br>**Sel.=95%**<br>(c) Conc.(SM1 in AcOH)=0.5M<br>*T*=20 °C/20-30 °C<br>*M-ratio*(N/SM1)=1.15<br>*M-ratio*(S/SM1)=1.15<br>**Isolated yield=82%**<br>**HPLC purity=99+%**<br>**Throughput=5.6 g/h**	Hastelloy C22 chip microreactor+PFA tubular reactor(OD 1/8 inch, ID 0.8 mm)
Hart, T. et al (2020) [23]	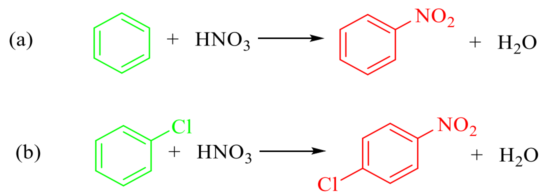			
	N/A	Aromatic compounds<br>/<br>(a)HNO_3-H_2SO_4<br>(b)Fuming HNO_3	**N/A**<br>(a) Solvent-free<br>Q_{SM}=0.23 mL/min<br>Q_{NR}=0.23 mL/min<br>*t*=5 min<br>*T*=80 °C<br>*P*=80 psi<br>**Yield=87%**<br>**Throughput=16.6 g/h**<br>(b) Solvent-free<br>Q_{SM}=64.6 μL/min<br>Q_{HNO3}=85.4 μL/min<br>*t*=10 min<br>*T*=65 °C<br>**Yield=96%**<br>**Throughput=5.7 g/h**	Chip reactor (Low-iron glass plates coated in a 0.13 mm fluorinated ethylene propylene film)
Sagmeister, P. et al (2020) [24]	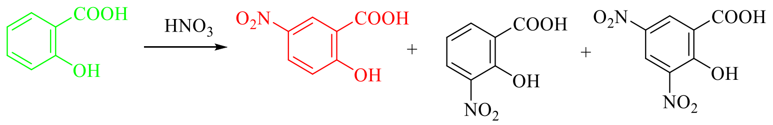			

	**H_2SO_4**<br>Conc.(SM)=0.5M (95%H_2SO_4)<br>Conc.(HNO_3)=0.6M (95%H_2SO_4)<br>*RT*=8.6-18.7 s<br>*M-ratio*(N/SM)=0.8-2<br>*T*=0-35 °C	Aromatic compounds / HNO_3-H_2SO_4	**DoE**<br>N/A	Hastelloy (C-276) chip microreactor (reactor volume for nitration 343μL)
Zhao, S. N. et al (2019) [25]	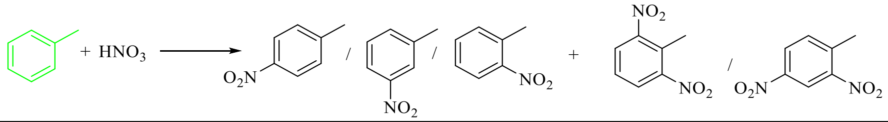			
	**Solvent-free**<br>*T*=5-45 °C<br>Q_{total}=1.5-4.5 mL/min<br>H_2SO_4/HNO_3/H_2O= 68.11/21.89/10 wt%<br>*M-ratio*(HNO_3/SM)=1.05<br>Channel size=0.6/1.0 mm<br>Ultrasound power=0/50 W<br>Ultrasound transmission medium=water/50 vol% aq. ethylene glycol	Aromatic compounds / HNO_3-H_2SO_4	**OFAT**<br>N/A	Ultrasonic tubular reactor(ID.0.6mm/1.0 mm)
Zharkov, M. N. et al (2019) [26]	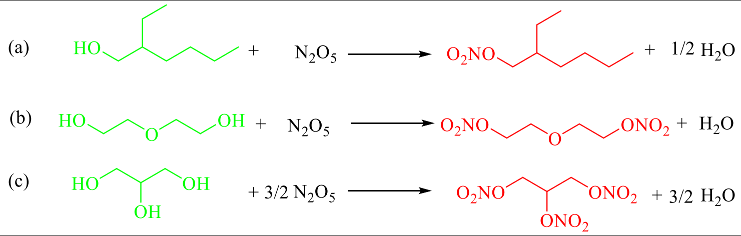			
	**1,1,1,2-tetrafluoroethane (R134a)**<br>(a) *t*=25-100 s; *T*=20 °C; *P*=10 bar<br>Q_{SM}=0.0565-0.2262mL/min<br>Q_{N2O5}=0.5190-2.1740mL/min<br>Reactor volume=0.25-1 mL<br>*M-ratio*(N_2O_5/SM)=1.0-1.5<br>**(b)** *t*=13/50 s, T=20 °C<br>*P*=10 bar, Q_{SM}=0.0496mL/min<br>Q_{N2O5}=1.151mL/min<br>Reactor volume=0.5/2 mL<br>*M-ratio*(N_2O_5/SM)=2.2<br>(c) *t*=4-50 s; *T*=20 °C; *P*=10 bar<br>Q_{SM}=0.0260-0.0780mL/min<br>Q_{N2O5}=1.174-3.522mL/min<br>Reactor volume=0.25-1 mL<br>*M-ratio*(N_2O_5/SM)=3.3-3.6	Aliphatic compounds / N_2O_5	**OFAT**<br>Solvent R134a<br>(a) *t*=25-100 s; *T*=20 °C;<br>*P*=10 bar<br>Q_{SM}=0.0565-0.2262mL/min<br>Q_{N2O5}=0.5190-2.1740mL/min<br>Reactor volume =0.25-1 mL<br>*M-ratio*(N_2O_5/SM)= 1.0-1.5<br>**Conv.=87%~100%**<br>**(b)** *t*=13/50 s, *T*=20 °C<br>*P*=10 bar,<br>Q_{SM}=0.0496mL/min<br>Q_{N2O5}=1.151mL/min<br>Reactor volume=0.5/2 mL<br>*M-ratio*(N_2O_5/SM)=2.2<br>**Conv.=90/99%**<br>**Sel.=100%**<br>(c) *t*=4-50 s; *T*=20 °C;<br>*P*=10 bar<br>Q_{SM}=0.0260-0.0780mL/min<br>Q_{N2O5}=1.174-3.522 mL/min<br>Reactor volume=0.25-1 mL<br>*M-ratio*(N_2O_5/SM)= 3.3-3.6<br>**Conv.=96~100%**<br>**Sel.=75~96%**	Steel tubular reactor(ID 0.03 inch)

Russo, D. et al (2019) [27] Russo, D. et al (2017) [28]	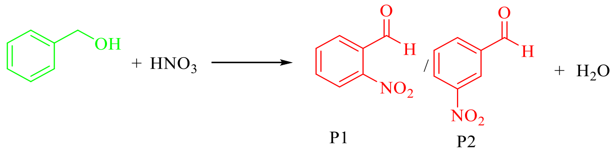			
	**Solvent-free**<br>x_n=0.080-0.450<br>x_s=0.270-0.450<br>T=45-68 °C<br>C_{SM}=0.05-0.5 M<br>Q_{MA}=0.20-9.99 mL/min<br>Q_{SM}=1.029-103.0 μL/min<br>V_R=1.5-4.5 mL	Aromatic compounds / HNO_3-H_2SO_4	**Kinetic modeling (for P1)**<br>Solvent-free<br>x_n=0.35; x_s=0.45; T=68 °C<br>**Yield $_{av}$(P1)=41.6%**<br>**SPY(P1)=0.33g·L^{-1}·s^{-1}(C_{SM}=0.05M)**<br>**SPY(P1)=3.29g·L^{-1}·s^{-1}(C_{SM}=0.5M)**<br>**Kinetic modeling (for P2)**<br>Solvent-free<br>x_n=0.130; x_s=0.318;<br>T=68 °C<br>C_{SM}=0.05 M<br>**Yield $_{av}$(P2)=96%**<br>**SPY(P2)=0.12g·L^{-1}·s^{-1}**	Commercial glass chip microreactor (Little Things Factory XXL-ST-04)
Sharma, Y. et al (2018) [29]	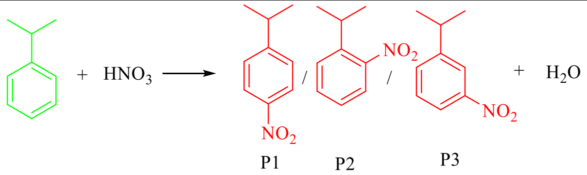			
	**Solvent-free**<br>t=2.64-10 min<br>T=0-40 °C<br>*M-ratio*(N/SM)=1-4	Aromatic compounds / Fuming HNO_3	**OFAT**<br>Solvent-free<br>t=6.4 min<br>T=10 °C<br>*M-ratio*(N/SM)=4<br>**Yield(P1/P2/P3) =79%/20%/1%**	AMaR1 micromixer+316 SS tubular reactor (OD 1/8 inch)
Rakshit, S. et al (2018) [30]	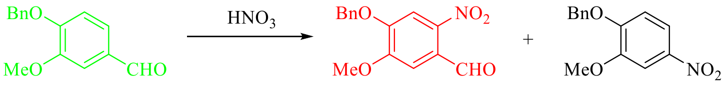			
	**Sulfolane**<br>t=3.4-11.3 min<br>T=35-45 °C<br>*M-ratio*(N/SM)=8.0-8.7	Aromatic compounds / Fuming HNO_3	**OFAT**<br>Solvent sulfolane<br>t=8.5 min<br>T=45 °C<br>*M-ratio*(N/SM)=8.7<br>**Yield=~70%**	Hastelloy(C22) tubular reactor(ID 1.8 mm)
Wen, Z. H. et al (2017) [31]	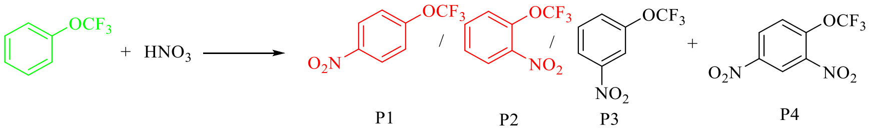			
	**Solvent-free**<br>*M-ratio*(N/SM)=0.85-1.00<br>*M-ratio*(S/N)=4<br>Water content φ= 88~98 wt%<br>t=4-16 s<br>T=273-293 K<br>Q_{total}=1.0-3.5 mL/min(for microchannel reactor)<br>Q_{total}=1.0-9.0 mL/min(for micro packed-bed reactor)	Aromatic compounds / HNO_3-H_2SO_4	**OFAT**<br>Solvent-free<br>*M-ratio*(N/SM)=1.1<br>*M-ratio*(S/N)=4<br>Water content φ=97 wt%<br>T=273 K<br>Q_{or}=0.4 mL/min<br>Q_{aq}=0.9 mL/min<br>**Conv.=99.6%**<br>**Sel.(P1/P2/P3/P4)= 90.97%/7.26%/0.08%/ 1.04%**	Microchannel reactor (SS tubular reactor, ID 0.6 mm); micro packed -bed reactor (tubular reactor, ID 0.6 mm +packed tubular reactor, ID 6 mm(packed with quartz sand microparticles))

Cantillo, D. et al (2017) [32]	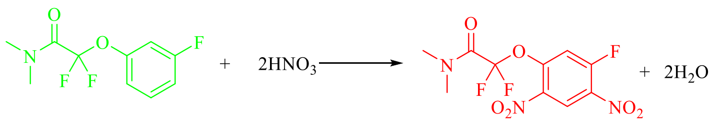			
	**Solvent-free**<br>Mixing Mode = T-mixer /T-mixer(Sonicated)/ Static glass mixer (IV 1.5mL, Uniqsis)<br>*M-ratio*(N/SM)=2.5<br>Conc.(HNO_3 in Oleum)= 5M<br>*T*=60/(rt+60) °C<br>*t*=0-31 min	Aromatic compounds<br>/<br>100%HNO_3-Oleum	**OFAT**<br>Solvent-free<br>*M-ratio*(N/SM)=2.5<br>Conc.(HNO_3 in Oleum)= 5M<br>*T*=60 °C<br>*t*=22 min<br>**Yield=93%**<br>**Isolated Yield=92%**	T-mixer/T-mixer(Sonicated)/Static glass mixer(IV 1.5mL)+PFA tubular reactor(ID 0.8mm, OD1.59mm)
Li, L. et al (2017) [33]<br>33	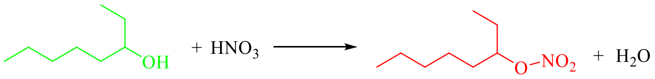			
	**Solvent-free**<br>*T*=286-307 K<br>*t*=3-10 s<br>Water content=3%-10%<br>*M-ratio*(N/SM)=0.7-1.6<br>*M-ratio*(S/N)=2	Aliphatic compounds<br>/<br>HNO_3-H_2SO_4	**OFAT**<br>Solvent-free<br>*T*=298 K<br>*t*=10 s<br>Water content=3%<br>*M-ratio*(N/SM)=1.54<br>*M-ratio*(S/N)=2<br>**Conv.=99+%**<br>**Sel.=99+%**	SS tubular reactor(ID 0.6 mm)
Chentsova, A. et al (2016) [34]	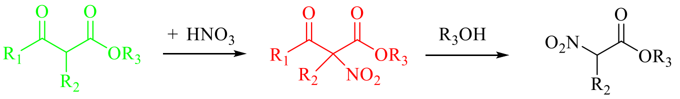			
	**Dichloromethane(DCM)**<br>**SM(α-acetylbutyrolactone)**<br>Conc.(SM)=0.36 M<br>*M-ratio*(N/SM)=1.2-1.4<br>*M-ratio*(S/N)=4-10<br>*t*=28-54 s<br>*T*=0-15 °C	Aliphatic compounds<br>/<br>HNO_3-H_2SO_4	**OFAT**<br>Solvent DCM<br>Conc.(SM)=0.36 M<br>*M-ratio*(N/SM)=1.4<br>*M-ratio*(S/N)=7.8<br>*t*=54 s<br>*T*=10 °C<br>**Conv.=95+%**<br>**Yield=80%**<br>**Isolated Yield=78%**	ETFE T-mixers+PTFE tubular reactor(ID 0.76 mm, OD 1.59 mm)
Yu, Z. et al (2016) [35]	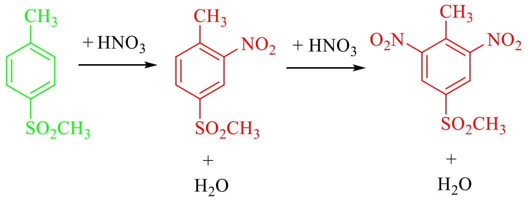			
	**H_2SO_4**<br>**Isothermal Mode**<br>***Mononitration***<br>*M-ratio*(N/SM)=1.05-1.2<br>*M-ratio*(S/N)=1.67-8.33<br>*t*=6-60 s<br>*T*=40-100 °C<br>Conc.(H_2SO_4)=70-98 wt%<br>LHSV=60-600 h^{-1}<br>***Dinitration***<br>Conc.(H_2SO_4)=80 wt%<br>*M-ratio*(N/SM2)=1.2<br>*M-ratio*(S/N)=6.25<br>LHSV=240 h^{-1}<br>*t*=5-60 s<br>*T*=40-120 °C<br>**Adiabatic Mode**<br>Conc.(H_2SO_4)=80 wt%<br>*M-ratio*(N/SM)=1.2	Aromatic compounds<br>/<br>Fuming HNO_3-H_2SO_4	**OFAT**<br>Solvent H_2SO_4<br>**Isothermal Mode**<br>***Mononitration***<br>Conc.(H_2SO_4)=80 wt%<br>*t*=15 s<br>*T*=80 °C<br>LHSV=240 h^{-1}<br>*M-ratio*(N/SM2)=1.2<br>*M-ratio*(S/N)=6.25<br>**Yield=98%**<br>**Purity=99%**<br>**Adiabatic Mode**<br>Conc.(H_2SO_4)=80 wt%<br>*M-ratio*(N/SM)=1.2<br>*M-ratio*(S/N)=2.9<br>*t*=5 s<br>*T*=adiabatic<br>**Yield=98%**	**Isothermal Mode**<br>SS 316 T-mixer (ID, 1.5 mm)+SS316 tubular reactor (ID 3mm, OD 5mm)<br>**Adiabatic Mode**<br>PTFE tubular reactor(ID 3mm)

	M-ratio(S/N)=2.5-7.5<br>t=0-8 s<br>T=adiabatic		**Purity=99%**	
Zhang, C.Y. et al (2016) [36]	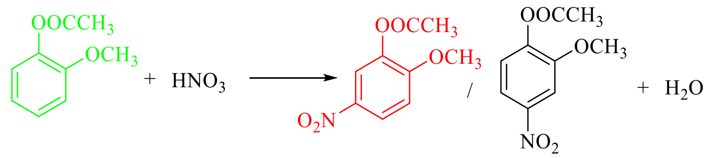			
	**HAc**<br>t=0-2 min<br>T=100-130 °C<br>*M-ratio*(N/SM)=1-4<br>Conc.(HNO_3 in Mixed)=20%-100%<br>P=0.7 MPa	Aromatic compounds<br>/<br>Fuming HNO_3-HAc	**OFAT**<br>Solvent HAc<br>t=2 min<br>T=120 °C<br>*M-ratio*(N/SM)=2.6<br>Conc.(HNO_3)=40%<br>P=0.7 MPa<br>**Yield=90.7%**	Tubular reactor(ID 2 mm)
Zuckerman, N. B. et al (2015) [37]	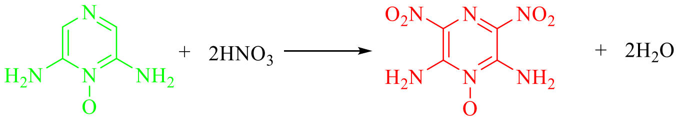			
	**Oleum**<br>T=10-40 °C<br>t= 6-10 min<br>t_{chip}=8-60 min<br>*M-ratio*(NO_2^+/SM)=2.2-4.9	Aromatic compounds<br>/<br>90% HNO_3-Oleum & KNO_3-Oleum	**OFAT**<br>Solvent oleum<br>*M-ratio*(NO_2^+/SM)=3.8<br>t=9 min<br>**Yield=49%**	Glass chip reactor/ETFE tubular reactor/Glass bead packed column
Raimondi, N. D. M. et al (2015) [38]	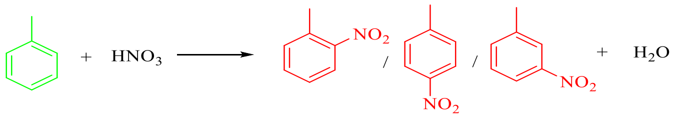			
	**Solvent-free**<br>Acid strength=0.75-0.80<br>T=23-35 °C<br>t=40-50 s<br>Q_{SM}=0.9-1.1 L/h<br>Q_{MA}=1.4-1.8 L/h<br>*M-ratio*(SM/HNO_3)=1.5	Aromatic compounds<br>/<br>HNO_3-H_2SO_4	**OFAT**<br>Solvent-free<br>Acid strength=0.80<br>T=27 °C<br>t=50 s<br>*M-ratio*(SM/HNO_3)=1.5<br>Conv.=33.7%<br>Sel.=95.1%<br>P1/P2/P3=54.8/40.5/4.6	SiC heat exchanger chip reactor (square section meandering channel, 2mm in depth)
Tibhe, J. et al (2014) [39]	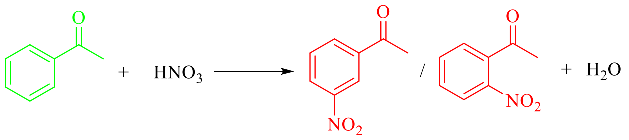			
	**H_2SO_4**<br>T=0-25 °C<br>t= 3-10 min<br>SM/H_2SO_4(Q_{SM})=1:2.58 v/v<br>HNO_3/H_2SO_4(Q_{MA})=1:1.5 v/v<br>Q_{SM}/Q_{MA}=0.8~2 v/v	Aromatic compounds<br>/<br>HNO_3-H_2SO_4	**OFAT**<br>Solvent H_2SO_4<br>T=10 °C<br>t=10 min<br>SM/H_2SO_4(Q_{SM})=1:2.5 w/v<br>HNO_3/H_2SO_4(Q_{MA})=1:1.5 v/v<br>Q_{SM}/Q_{MA}=1:1.66 v/v<br>**Conv.=100%**<br>**Yield=98.55%**	316L SS tubular reactor

Note: In the reaction scheme, the starting materials are marked in yellow, and the main products are labeled in red. Analysis methods follows convention: pure OFAT screening is termed OFAT; analyses incorporating DoE frameworks (even with OFAT elements) are classified as DoE.

NOMENCLATURE

A	pre-exponential factor
Ac_2O	acetic anhydride
ARC	accelerating rate calorimetry
C80	Calvet calorimeter C80
C_{NA}	the concentrations of NA
C_{NA}^0	the initial concentration of HNO_3
$C_{NO_2^+}$	the concentrations of NO_2^+
Conc.	concentration
Conv.	conversion
C_{SM}	the concentration of SM
C_{SM}^0	the initial concentrations of SM
CSTR	continuous stirred tank reactor
Da_{II}	second Damköhler number
DCM	dichloromethane
DCE	1,2-dichloroethane
DoE	design of Experiments
DSC	differential scanning calorimetry
E_a	activation energy
HAc	acetic acid
Ha	Hatta number
ΔH_D	heat of decomposition
k_2^*	the observed second-order reaction rate constant related to the NO_2^+
k_{obs}	the observed reaction rate constant based on HNO_3
LHSV	liquid hourly space velocity
M	the stoichiometric feed ratio of HNO_3 to SM
M_c function	represents the acidity of H_2SO_4
M/M	mole/mole
M-ratio (N/SM)	the molar ratio of HNO_3 to starting material
M-ratio (N/S)	the molar ratio of HNO_3 to H_2SO_4
MTSR	maximum temperature of the synthesis reaction
MTT	maximum temperature for technical reason
MW	molecule weight
n	a thermodynamic parameter that depends on the nitration substrate
NA	HNO_3
OB	oxygen balance
OFAT	one-factor-at-a-time

Q_A	the flow rate of acid
Q_{aq}	the flow rate of aqueous phase
Q_{MA}	the flow rate of mixed acid
Q_{or}	the flow rate of organic phase
Q_{SM}	the flow rate of SM
Q_{total}	total flow rate
R134a	1,1,1,2-tetrafluoroethane
RC1	reaction calorimeter
RT	residence time
R.T.	room temperature
Re	Reynolds number
Sel.	selectivity
SM	starting material
STY	space time yield
T	temperature
ΔT_{ad}	adiabatic temperature rise
T_{D24}	the initial temperature when TMR_{ad} equals 24 h
TMR_{ad}	time to maximum rate under adiabatic condition
TP	throughput
T_P	temperature of process
v/v	volume/volume
V-ratio (N/S)	the volume ratio of HNO_3/H_2SO_4
V_R	the volume of reactor
w_{AA}	the mass fraction of acetic anhydride
w/w	weight/weight
X	the conversion of SM
x_n	nitric acid molar fraction
x_s	sulfuric acid molar fraction
φ	*water content*
γ^*	the activity coefficients of the transition intermediate
$\gamma_{NO_2^+}$	the activity coefficients of NO_2^+
γ_{SM}	the activity coefficients of SM

REFERENCES

1. Wang, J. C.; Pan, Y.; Wang, Y. J.; Ni, L.; Leveneur, S. *Chem. Eng. Process.* **2024**, 204, 109934.
2. Cao, J. Y.; Hou, J.; Zhan, L. W.; Li, B. D. *J. Flow Chem.* **2024**, 14, 281-288.
3. Mittal, A. K.; Pathak, P.; Prakash, G.; Maiti, D. *Chem. - Eur. J.* **2023**, 29, e202301662.
4. Xu, F.; Chen, Z.; Ni, L.; Fu, G.; Liu, J.; Jiang, J. C. *Org. Process Res. Dev.* **2023**, 27, 2134-2145.
5. Guo, S.; Cao, J. Y.; Liu, M. Q.; Zhan, L. W.; Li, B. D. *Chem. Eng. Process.* **2023**, 183, 109239.
6. Guo, S.; Zhan, L.; Li, B. *Chem. Eng. J.* **2023**, 468, 143468.
7. Guo, S.; Zhan, L.; Li, B. *Chem. Eng. J.* **2023**, 477, 147011.
8. Mittal, A. K.; Prakash, G.; Pathak, P.; Maiti, D. *Chem. - Asian J.* **2023**, 18, e202201028.
9. Petho, B.; Szilágyi, G. B.; Mengyel, B.; Nagy, T.; Farkas, F.; Kátai-Fadgyas, K.; Volk, B. *Org. Process Res. Dev.* **2022**, 26, 1223-1235.
10. Guo, S.; Zhu, G. K.; Zhan, L. W.; Li B. D. *J. Flow Chem.* **2022**, 12, 327-336.
11. Fu, G.; Ni, L.; Wei, D.; Jiang J. C.; Chen Z. Q.; Pan, Y. *Process Saf. Environ. Prot.* **2022**, 160, 385-396.
12. Guo, S.; Zhu, G.; Zhan, L.; Li, B. D. *Chem. Eng. Res. Des.* **2022**, 178, 179-188.
13. Mule, G. M.; Kulkarni, S.; Kulkarni, A. A. *React. Chem. Eng.* **2022**, 7, 1671-1679.
14. Sacher, S.; Castillo, I.; Rehrl, J.; Sagmeister, P.; Lebl, R.; Kruisz, J.; Celikovic, S.; Sipek, M.; Williams, J. D.; Kirschneck, D.; Kappe, C. O.; Horn, M. *Chem. Eng. Res. Des.* **2022**, 177, 493-501.
15. Lan, Z.; Lu, Y. *J. Flow Chem.* **2021**, 11, 171-179.
16. Sagandira, M. B.; Sagandira, C. R.; Watts, P. *J. Flow Chem.* **2021**, 11, 193-208.
17. Cardinal-David, B.; Harper, K. C.; Verma, A.; Hanna, D.; Caspi, D. D.; Vitale, C.; Bien, J. T.; Wang, Z.; Diwan, M. *Org. Process Res. Dev.* **2021**, 25, 2473-2481.

18. Hussain, A.; Sharma, M.; Patil, S.; Acharya, R. B.; Kute, M.; Waghchaure, A.; Kulkarni, A. A. *J. Flow Chem.* **2021**, 11, 611-624.
19. Sharma, M.; Acharya, R. B.; Kulkarni, A. A. *Chem. Eng. Technol.* **2019**, 42, 2241-2251.
20. Kyprianou, D.; Berglund, M.; Emma, G.; Rarata, G.; Anderson, D.; Diaconu, G.; Exarchou, V. *Molecules* **2020**, 25, 3586.
21. Chen, P.; Shen, C.; Qiu, M.; Wu, J.; Bai, Y. J.; Su, Y. H. *J. Flow Chem.* **2020**, 10, 207-218.
22. Köckinger, M.; Wyler, B.; Aellig C.; Roberge, D. M.; Hone, C. A.; Kappe, C. O. *Org. Process Res. Dev.* **2020**, 24, 2217-2227.
23. Hart, T.; Schultz, V. L.; Thomas, D.; Kulesza, T.; Jensen, K. F. *Org. Process Res. Dev.* **2020**, 24, 2105-2112.
24. Sagmeister, P.; Poms, J.; Williams, J. D. D.; Kappe, C. O. *React. Chem. Eng.* **2020**, 5, 677-684.
25. Zhao, S. N.; Yao, C. Q.; Zhang, Q.; Chen, G. W.; Yuan, Q. *Chem. Eng. J.* **2019**, 374, 68-78.
26. Zharkov, M. N.; Arabadzhi, S. S.; Kuchurov, I. V.; Zlotin, S. G. *React. Chem. Eng.* **2019**, 4, 1303-1308.
27. Russo, D.; Tomaiuolo, G.; Andreozzi, R.; Guido, S.; Lapkin, A. A.; Di Somma, I. *Chem. Eng. J.* **2019**, 377, 120346.
28. Russo, D.; Di Somma, I.; Marotta, R.; Tomaiuolo, G.; Andreozzi, R.; Guido, S.; Lapkin, A. A. *Org. Process Res. Dev.* **2017**, 21, 357-364.
29. Sharma, Y.; Nikam, A. V.; Kulkarni, A. A. *Org. Process Res. Dev.* **2018**, 23, 170-176.
30. Rakshit, S.; Lakshminarasimhan, T.; Guturi, S.; Kanagavel, K.; Kanusu, U. R.; Niyogi, A. G.; Sidar, S.; Luzung, M. R.; Schmidt, M. A.; Zheng, B.; Eastgate, M. D.; Vaidyanathan, R. *Org. Process Res. Dev.* **2018**, 22, 391-398.
31. Wen, Z. H.; Jiao, F.J.; Yang, M.; Zhao, S. N.; Zhou, F.; Chen, G. W. *Org. Process Res. Dev.* **2017**, 21, 1843-1850.
32. Cantillo, D.; Wolf, B.; Goetz, R.; Kappe, C. O. *Org. Process Res. Dev.* **2017**, 21, 125-132.
33. Li, L.; Yao, C. Q.; Jiao, F. J.; Han, M.; Chen, G. W. *Chem. Eng. Process.* **2017**, 117, 179-185.

34. Chentsova, A.; Ushakov, D. B.; Seeberger, P. H.; Gilmore, K. *J. Org. Chem.* **2016**, 81, 9415-9421.
35. Yu, Z.; Zhou, P.; Liu, J.; Wang, W. Z.; Yu, C. M.; Su, W. K. *Org. Process Res. Dev.* **2016**, 20, 199-203.
36. Zhang, C.Y.; Zhang, J. S.; Luo G. S. *J. Flow Chem.* **2016**, 6, 309-314.
37. Zuckerman, N. B.; Shusteff, M.; Pagoria, P. F.; Gash, A. E. *J. Flow Chem.* **2015**, 5, 178-182.
38. Raimondi, N. D. M.; Olivier-Maget, N.; Gabas, N.; Cabassud, M.; Gourdon, C. *Chem. Eng. Res. Des.* **2015**, 94, 182-193.
39. Tibhe, J.; Sharma, Y.; Joshi, R. A.; Joshi, R. R.; Kulkarni, A. A. *Green Process. Synth.* **2014**, 3, 279-285.
